# A Follow-Up to the Geographical Distribution of *Anopheles* Species in Malaria-Endemic and Non-Endemic Areas of Honduras

**DOI:** 10.3390/insects13060548

**Published:** 2022-06-15

**Authors:** Denis Escobar, Osman Archaga, Allan Reyes, Adalid Palma, Ryan T. Larson, Gissella M. Vásquez, Gustavo Fontecha

**Affiliations:** 1 Microbiology Research Institute, Universidad Nacional Autónoma de Honduras, Tegucigalpa 11101, Honduras; denis.escobar@unah.edu.hn (D.E.); osman.archagav@gmail.com (O.A.); 2 Unidad de Entomología, Región Sanitaria de Gracias a Dios, Secretaría de Salud de Honduras, Puerto Lempira, Gracias a Dios 33101, Honduras; arfrangar@yahoo.com; 3 Vysnova Partners, Inc., Landover, MD 20785, USA; adalid1996.31@hotmail.com; 4 Department of Entomology, U.S. Naval Medical Research Unit No. 6 (NAMRU-6), Bellavista 07006, Peru; ryan.t.larson6.mil@mail.mil (R.T.L.); gissella.m.vasquez.ln@mail.mil (G.M.V.)

**Keywords:** *Anopheles* spp., Honduras, genetic diversity, *COI* gene

## Abstract

**Simple Summary:**

Malaria is a tropical disease caused by parasites of the genus *Plasmodium*. The parasite is transmitted to humans through the bite of the female mosquito *Anopheles*. Honduras is close to the goal of eliminating malaria, but the region called La Moskitia continues to concentrate almost all of the country’s malaria cases. One of the key factors in achieving malaria elimination is a thorough understanding of the mosquito vectors that transmit the disease. There are few studies related to malaria vectors in Honduras. This study aims to contribute to knowing which are the species of vector mosquitoes, mainly in the Department of Gracias a Dios and in other departments in which cases of malaria occur, in addition to describing molecularly for the first time the anophelines of the Bay Islands. The most abundant species found here were *Anopheles albimanus*, but seven other species were also identified, some of which may contribute to parasite transmission.

**Abstract:**

*Anopheles* species are the vectors of malaria, one of the diseases with the greatest impact on the health of the inhabitants of the tropics. Due to their epidemiological relevance and biological complexity, monitoring of anopheline populations in current and former malaria-endemic areas is critical for malaria risk assessment. Recent efforts have described the anopheline species present in the main malaria foci in Honduras. This study updates and expands knowledge about *Anopheles* species composition, geographical distribution, and genetic diversity in the continental territory of Honduras as in the Bay Islands. Outdoor insect collections were carried out at 25 sites in eight municipalities in five departments of Honduras between 2018 and 2021. Specimens were identified using taxonomic keys. Partial COI gene sequences were used for molecular species identification and phylogenetic analyses. In addition, detection of *Plasmodium* DNA was carried out in 255 female mosquitoes. Overall, 288 *Anopheles* mosquitoes were collected from 8 municipalities. Eight species were morphologically identified. *Anopheles albimanus* was the most abundant and widely distributed species (79.5%). A subset of 175 partial COI gene sequences from 8 species was obtained. Taxonomic identifications were confirmed via sequence analysis. *Anopheles albimanus* and *An. apicimacula* showed the highest haplotype diversity and nucleotide variation, respectively. Phylogenetic clustering was found for *An. argyritarsis* and *An. neomaculipalpus* when compared with mosquitoes from other Neotropical countries. *Plasmodium* DNA was not detected in any of the mosquitoes tested. This report builds upon recent records of the distribution and diversity of *Anopheles* species in malaria-endemic and non-endemic areas of Honduras. New *COI* sequences are reported for three anopheline species. This is also the first report of *COI* sequences of *An. albimanus* collected on the island of Roatán with apparent gene flow relative to mainland populations.

## 1. Introduction

Honduras has maintained a substantial reduction in malaria cases in the last two decades. The current number of cases has been reduced by 97% relative to the 35,125 cases in 2000 [1]. The department of Gracias a Dios (La Moskitia region) accounts for more than 96% of the country’s cases, and 99% of the cases are due to *Plasmodium falciparum*. Puerto Lempira in Gracias a Dios is the main focus of malaria in Honduras. In 2020, 12 active malaria foci were detected in the country, mainly in Gracias a Dios, and residual foci in the departments of Yoro, Bay Islands, Colón, and El Paraíso (personal communication, Honduras Ministry of Health). The control measures implemented in the country to achieve the goal of eliminating malaria by 2030 include, among others, entomological surveillance and control of vector mosquito populations [2].

*Anopheles albimanus* is the most abundant and widespread species in the country [3]. The genus *Anopheles* (Culicidae: Anophelinae) includes almost 500 species grouped into at least 7 subgenera [4,5]. In Honduras, 13 species of the genus *Anopheles* belonging to 3 subgenera have been identified: *Anopheles Anopheles* (*Anopheles crucians*, *Anopheles pseudopunctipennis*, *Anopheles vestitipennis*, *Anopheles punctimacula*, *Anopheles neomaculipalpus*, *Anopheles apicimacula*, *Anopheles gabaldoni*, and *Anopheles grabhamii*), *An*. *Nyssorhynchus* (*Anopheles albimanus*, *Anopheles darlingi*, *Anopheles argyritarsis*, and *Anopheles albitarsis*), and *An*. *Kerteszia* (*Anopheles neivai*). Four of these species are considered dominant vector species of human malaria: *Anopheles* (*Nys.*) *albimanus*, *An.* (*An.*) *pseudopunctipennis*, *An*. (*Nys.*) *darlingi*, and *An*. (*Nys.*) *albitarsis*) [6,7,8].

Each anopheline species displays different bionomic traits that are relevant to malaria epidemiology, such as feeding preference, endophagic/exophagic behaviour, resting behaviour, larval habitat preference, etc. [9,10,11,12,13]. For instance, *An. albimanus* is considered a generalist vector, with opportunistic feeding preferences and capable of colonising a wide range of habitats [10,14]. On the other hand, *An. darlingi* requires high levels of humidity to develop its life cycle [15]; nevertheless, both species are considered dominant malaria vectors [7,8]. Climate change is predicted to have a direct effect on the distribution and dynamics of human malaria vectors [16,17]. Consequently, continuous monitoring of changes in the distribution of vector species and their bionomic characteristics is relevant for decision makers in each country to efficiently channel resources for malaria control.

Identification of anopheline species for entomological surveillance purposes requires in-depth morphological knowledge and considerable experience. However, the genus *Anopheles* includes several cryptic species [18], and it is not always possible to morphologically identify all species, particularly in geographical areas cohabited by sibling species belonging to a taxonomic complex [19,20]. To overcome the limitations of taxonomic keys, sequencing of the *cytochrome c oxidase* 1 (COI) gene has proven to be a valuable tool due to its low level of recombination, absence of introns, and haploidy [21,22,23].

This research was built on a previous study [3] by assessing the species composition, geographical distribution, and genetic diversity of *Anopheles* species in five departments of Honduras, including the Bay Islands, using conventional taxonomic keys and a mitochondrial genetic marker (*COI*) and will further contribute to malaria entomological risk assessment in Honduras. Molecular screenings of mosquitoes infected with *Plasmodium* spp. sporozoites were also carried out.

## 2. Materials and Methods

### 2.1. Study Sites, Mosquito Collection, and Morphological Identification

**Figure 1:**
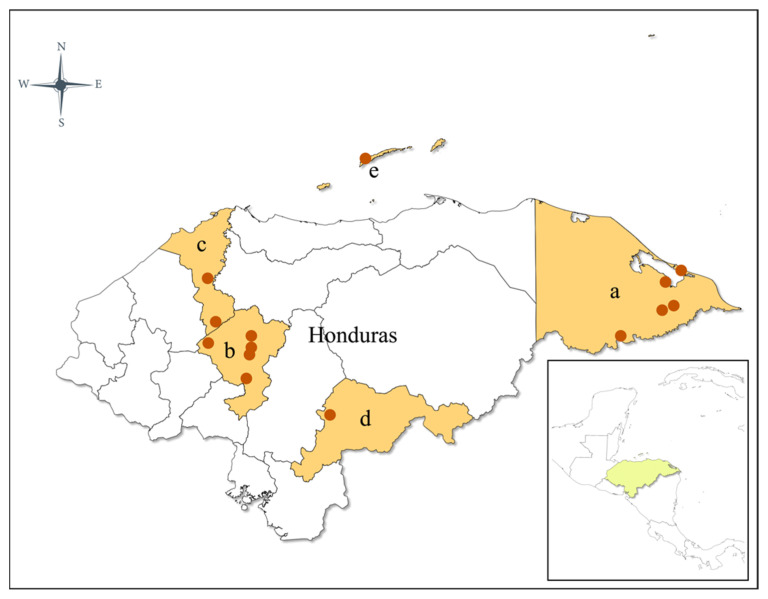
Map of Honduras showing study sites in five departments where entomological collections were conducted: (**a**) Gracias a Dios; (**b**) Comayagua; (**c**) Cortés; (**d**) El Paraíso; (**e**) Bay Islands.

Insect collections were carried out between 2018 and 2021 at 25 sites in 8 municipalities and 5 departments of Honduras (Figure 1, Table 1). Collection site coordinates were recorded using a handheld GPS. The sites in the municipalities of Gracias a Dios, Roatán, and El Paraíso are endemic to malaria. No malaria cases were reported in the municipalities of Comayagua and Cortés during the study collection period. Collection sites were all rural areas and included distinct climates. Gracias a Dios, Bay Islands, and Cortés are very humid, coastal tropical ecosystems less than 550 m above sea level (masl), while Comayagua and El Paraíso are mountainous subtropical regions, with heights above 550 masl and drier ecosystems. The average temperature varies between 25 °C and 33 °C, and the relative humidity ranges from 40% to 91% in all sites depending on the season of the year.

Ecological differences among collection sites are considerable; the economic activities are also diverse. Agriculture and livestock prevail in all the collection sites; however, fishing and activities related to tourism are relatively more common in Gracias a Dios and Bay Islands, respectively. Collections sites were selected based on historical and current malaria case reports. Several mosquito collection methods were employed (Supplementary Table S1). All mosquito captures were conducted outdoors in both peridomicile and extradomicile areas. Overall, adult mosquito traps (CDC light traps, Shannon-type tent traps), were installed between 10 and 50 m away from the selected dwellings (2 to 5 per locality) and operated between 18:00 and 06:00 h, for two or three nights per collection site. In addition, mosquitoes resting outdoors were collected with manual aspirators between 18:00 and 21:00 h. The use of various collection methods allowed us to increase the probability of collecting different species of anophelines during the period from 18:00 to 06:00 h. Mosquitoes were placed in conic microtubes with silica gel and transported to Tegucigalpa city for species morphological identification. All specimens were identified morphologically under a stereoscope using keys for anophelines of Central America and Mexico [24]. Each mosquito was stored individually in 1.5 mL microtubes at −20 °C for subsequent molecular analyses.

### 2.2. DNA Extraction, COI Gene Amplification, and Sequencing

DNA was extracted from each specimen collected following DNeasy Blood and Tissue Kit^®^ (QIAGEN, Hilden, Germany) protocol. First, the head and thorax were dissected for each mosquito. Single maceration was carried out with a pestle in a 1.5 mL conical tube following the manufacturer’s instructions. Overnight lysis at 56 °C was carried out. DNA was eluted in 150 μL of elution buffer and stored at −20 °C until further use. Molecular analyses were performed on *Anopheles* mosquitoes to identify or confirm species using a barcoding approach, which also allowed genetic variation within species and between species to be calculated. The *cytochrome c oxidase 1* gene (*COI*) was amplified with the following primers: LCO1490 (5′—GGT CAA CAA ATC ATA AAG ATA TTG G—3′) and HCO2198 (5′-TAA ACT TCA GGG TGA CCA AAA ATC A—3′) [25].

Reactions were carried out as described previously [3] in a volume of 50 μL, with 25 μL of Taq Master Mix 2× (Promega, Madison, WI, USA), 2.0 μL of each primer (10 μM), 2 μL of acetylated bovine albumin (BSA) (10 mg/mL), 4 μL of DNA, and nuclease-free water. The PCR program was as follows: 1 cycle at 95 °C for 10 min, 37 cycles at 94 °C for 1 min, 48 °C for 1 min, 72 °C for 1 min, and 1 cycle at 72 °C for 7 min. Some mosquito specimens that could not be amplified as described above were amplified using LCO1490 and a reverse primer described by Kumar et al. [26] (5′—AAA AAT TTT AAT TCC AGT TGG AAC AGC—3′); under the following conditions: 25 μL of Taq Master Mix 2× (Promega, Madison, WI, USA); 1 μL of each primer (10 μM); 2 μL of DNA; and 21 μL of nuclease-free water. The cycling conditions were as follows: 1 cycle at 95 °C for 5 min, 5 cycles at 94 °C for 40 s, 45 °C for 1 min, 1 cycle at 72 °C for 1 min, 37 cycles at 94 °C for 1 min, 54 °C for 1 min, 72 °C for 90 s, and a final extension step at 72 °C for 10 min. PCR products of approximately 700 bp were separated via electrophoresis in 1% agarose gels with ethidium bromide.

The amplification products were sequenced on both strands using the same primers that were also used for the PCR. Sequencing services were provided by Psomagen^®^ (www.psomagen.com, Rockville, MD, USA). The sequences were edited with the Geneious^®^ 9.1.7 software and deposited into the NCBI GenBank.

### 2.3. Sequence Analyses

Partial sequences of the *COI* gene were analysed together for the eight species, and separately for each species. Sequences were aligned with the MUSCLE algorithm of the Geneious^®^ 9.1.7 software. The length of the nucleotide sequences, the number and percentage of identical sites, and the pairwise % identity, were calculated. The percentage of identical bases within and between species was calculated. The number of haplotypes was calculated according to the nucleotide sequences. Nucleotide sequences were translated using the correct open reading frame (ORF) and using the invertebrate mitochondrial genetic code. The amino acid length of each polypeptide and the number of different haplotypes were calculated. MEGA v10.0 software [27] with 500 bootstrap replicates was used to calculate the overall mean diversity (π) using the maximum composite likelihood substitution method, and 95% as the site coverage cutoff.

The numbers of haplotypes (h) and haplotype diversity (Hd) were calculated for each species with DnaSP software v. 6.12.03 [28]. Alignment sequences were imported, and parameters were adjusted for mitochondrial DNA with genetic code for *Drosophila* mtDNA. Haplotype data were generated using the Roehl data file function and default parameters.

Phylogenetic trees were constructed using the sequences obtained in this study for *An. argyritarsis*, *An. apicimacula*, and *An. neomaculipalpus*, together with homologous sequences from individuals from other regions of the Americas and downloaded from GenBank. The remaining five species described in this study (*An. albimanus*, *An. crucians*, *An. vestitipennis*, *An. punctimacula*, and *An. pseudopunctipennis*) were not subjected to phylogenetic analyses with individuals from other countries because that analysis had already been described in a previous study [3]. Phylogenetic analyses used the Tamura–Nei distance model, the neighbour-joining method, and a bootstrap of 1000 replicates, with a sequence of *Culex nigripalpus* as an outgroup.

### 2.4. cox1 Gene PCR for Plasmodium Sporozoite Detection

To detect DNA from sporozoites of *Plasmodium* spp., 43 pools of DNA from 228 female mosquitoes visibly engorged or not were analysed. A total of 128 mosquitoes from Puerto Lempira, 38 from Comayagua, 28 from El Paraíso, 6 from Cortés, and 28 from Roatán were analysed. Across sites, 165 *An. albimanus*, 3 *An. apicimacula*, 18 *An. argyritarsis*, 10 *An. crucians*, 10 *An. neomaculipalpus*, 15 *An. pseudopunctipennis*, 2 *An. punctimacula*, and 5 *An. vestitipennis* were tested.

Using a stereoscope, the head and thorax of the specimens were separated, and DNA was extracted as described above. The detection of the parasite genome was based on the amplification of the *cytochrome oxidase I* (*cox1*) gene, as described by Echeverry et al. [29]. Briefly, reactions were carried out in a final volume of 25 µL containing 12.5 µL of Master Mix 2× (Promega Corp. Madison, WI, USA), 1 µL of each primer (COX1F 5’-AGA ACG AAC GCT TTT AAC GCC TG—3′/COX-IR 5´-TGW CCT ACC TGA AAT ATA GGT AAT TCT—3´) at a concentration of 10 µM, 9.5 µL of nuclease-free water, and 1 µL of genomic DNA 20–40 ng/µL). Mosquito DNA was analysed in pools [30] of 6 individuals per reaction. The amplification program included 1 cycle of 5 min at 94 °C, followed by 40 cycles of 1 min at 94 °C, 1 min at 62 °C, and 90 s at 72 °C, with a final extension of 10 min at 72 °C. The PCR products were separated via electrophoresis in 1% agarose gels with ethidium bromide. Positive and negative controls were included in all experiments. Any sample with a band of approximately 540 bp was considered positive.

To determine the lower limit of detection of the PCR, 10 consecutive serial decimal dilutions of the *Plasmodium* sp. culture number 04/176 were carried out. Dilutions included DNA concentrations from 35,000 IU/µL to 3.5 × 10^−6^. Samples were tested in triplicate, and each experiment included positive and negative controls. In addition, analyses were conducted to assess the ability of the PCR to detect DNA from a *Plasmodium* sp. positive sample when pooled with up to other 9 negative blood samples.

## 3. Results

### 3.1. Distribution of Anopheles Species

Of all the insects captured in the 8 municipalities, 288 anopheline mosquitoes were selected according to their morphological identification. Taxonomic identification revealed eight species: *Anopheles* (*Nyssorhynchus*) *albimanus* Wiedemann; *An.* (*Nys.*) *argyritarsis* Robineau-desvoidy; *An. (An.) crucians* Wiedemann; *An*. (*An*.) *neomaculipalpus* Curry; *An*. (*An*.) *vestitipennis* Dyar and Knab; *An*. (*An*.) *pseudopunctipennis* Theobald; *An*. (*An*.) *apicimacula* Dyar and Knab; and *An*. (*An*.) *punctimacula* Dyar and Knab.

**Figure 2:**
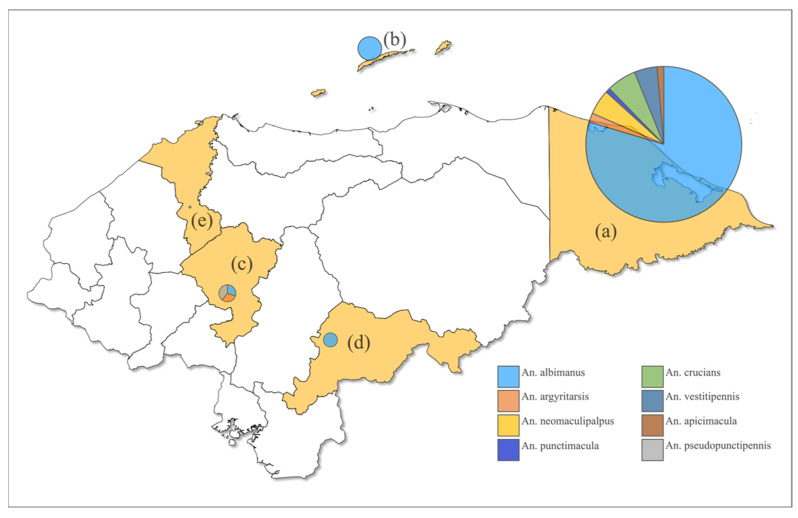
Map of Honduras showing pie charts with the proportion of *Anopheles* species collected at each department. Chart size is proportional to the number of specimens collected: (**a**) Gracias a Dios; (**b**) Bay Islands; (**c**) Comayagua; (**d**) El Paraíso; (**e**) Cortés.

The majority (79.5%) of the anophelines were identified as *An. albimanus*, followed distantly by *An. crucians* (4.5%) and *An. argyritarsis* (3.8%). The least frequent species were *An. apicimacula* (1.04%) and *An. punctimacula* (0.7%) (Table 2, Figure 2). *Anopheles albimanus* was found in all five departments, in six of eight localities, while *An*. *pseudopunctipennis* was described only in Comayagua. The greatest abundance of specimens and the greatest richness of species was found in the department of Gracias a Dios (73.3%).

### 3.2. Nucleotide Sequences and Diversity

A subset of 175 mosquitoes was sequenced and included specimens from all 8 species and all geographical regions. A total of 175 partial sequences of the mitochondrial *COI* gene were obtained. All sequences were deposited in GenBank under the following accession numbers: *An. albimanus* (OL473449—OL473511, OM366057—OM366082); *An. apicimacula* (OL473781—OL473784, OM366084—OM366086); *An. neomaculipalpus* (OL473750—0L473769); *An. argyritarsis* (OL471412—OL471427); *An. pseudopunctipennis* (OL473573—OL473586); *An. punctimacula* (OL473785—OL473786); *An. crucians* (OL514242—OL514261); and *An. vestitipennis* (OL515123—OL515128). This study is the first report of *COI* gene sequences for three anopheline species collected in Honduras (*An. argyritarsis*, *An. apicimacula*, and *An. neomaculipalpus*).

**Figure 3:**
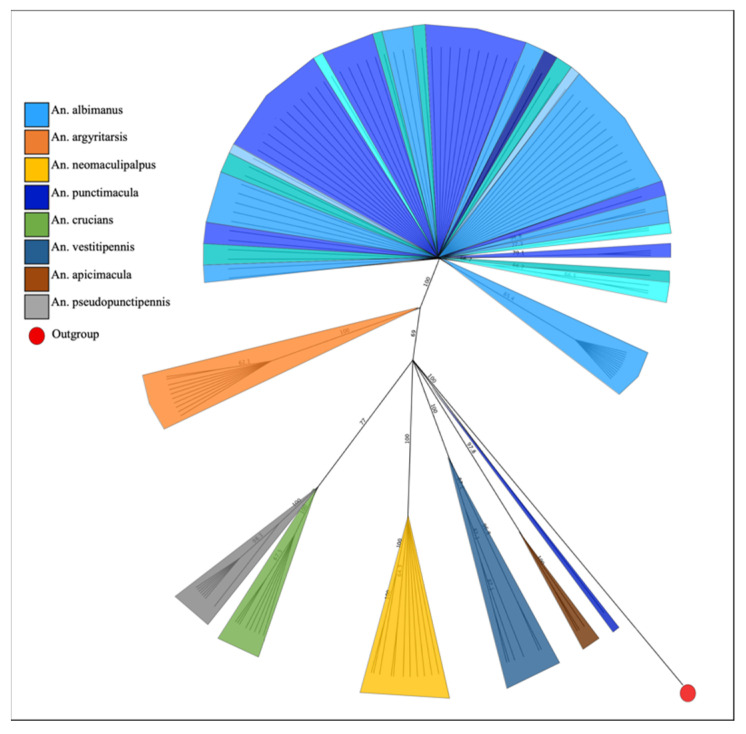
Phylogenetic cladogram of *COI* sequences of eight *Anopheles* species constructed using the neighbour-joining method with a bootstrap of 1000 replicates and Geneious 9.1.7 software. Shades of blue indicate different *An. albimanus* collection sites.

The percentage of intra- and interspecific identity for the eight species was non-overlapping, averaging 98.74% (94.16–100%) and 88.23% (85.47–91.1%), respectively. The sequences were analysed with the NCBI BLAST tool to confirm the taxonomic identification based on the morphological structures of the insects. The eight species were confirmed with identity percentages between 93.5% (*An. apicimacula*) and 100% (*An. crucians*), and 100% query coverage. The phylogenetic cladogram built from the sequences obtained for the eight species shows a clear and coherent separation into clusters (Figure 3).

Despite including only three sequences, *An. apicimacula* showed the highest intraspecies nucleotide variation (π = 0.04). On the other hand, the species with the lowest diversity was *An. neomaculipalpus* (π = 0.00), with 11 sequences. *Anopheles albimanus* showed the highest haplotype diversity and number of haplotypes (Table 3).

### 3.3. Phylogenetic Analysis

**Figure 4:**
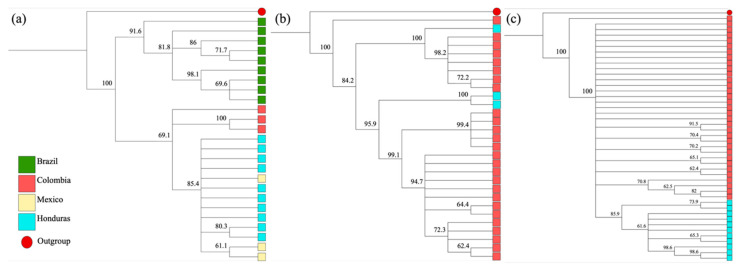
Dendrograms constructed using the neighbour-joining method and Geneious 9.1.7 software with a bootstrap of 1000 replicates: (**a**) *Anopheles argyritarsis*; (**b**) *An. apicimacula*; (**c**) *An. neomaculipalpus*.

Phylogenetic analyses were performed using three independent alignments with sequences from *An. argyritarsis*, *An. apicimacula*, and *An. neomaculipalpus* obtained in this study and homologous sequences downloaded from GenBank from anophelines collected in other countries. The analysis of *An. argyritarsis* included 10 sequences from Honduras and 15 sequences from Colombia (Acc. Nº HM022395, HM022396); Brazil (Acc. Nº KT762354, KU762348, KU671369, KT762357, MF381679, MZ389742, KT762357, MZ389741, MZ389743); and Mexico (Acc. Nº MT999181, MT999194, M7999170), with a size of 596 bp. The analysis of *An. apicimacula* included 3 sequences from Honduras and 26 sequences from Colombia with a size of 585 bp (Acc. Nº OM366084-6, KU900813-7, KF698866-72, MG701357-67). Finally, the analysis of *An. neomaculipalpus* included 11 sequences from Honduras and 33 sequences from Colombia (Acc. Nº OM366087, JX205125, MG701376-82, KU900755, KM592986, KF698843-64) with a size of 572 bp. The dendrogram obtained from the sequences of *An. argyritarsis* revealed an evident clustering, with high bootstrap indices, that separates individuals from Brazil and Colombia from individuals from Honduras/Mexico (Figure 4a). Similarly, *An. neomaculipalpus* mosquitoes from Honduras are separated from those from Colombia (Figure 4c). In the case of the dendrogram of *An. apicimacula*, no clusters were detected based on geographical origin (Figure 4b).

### 3.4. Plasmodium spp. DNA Detection

The *Plasmodium* spp. *cox1* gene was amplified in 43 pools of DNA from 228 female *Anopheles* comprising 8 species. *Plasmodium* spp. DNA was not detected in any of the 228 *Anopheles* mosquitoes tested.

## 4. Discussion

In this study, 288 anophelines from 5 departments of Honduras (Gracias a Dios, El Paraíso, Comayagua, Cortés, and Bay Islands) were identified through morphology and molecular biology. The relative abundance of mosquitoes was also described, and the absence of parasite DNA in the head/thorax of mosquitoes was reported. The results described here build upon a recent study published in 2020 [3], which included the taxonomic and molecular identification of 1320 *Anopheles* mosquitoes collected in 5 departments of Honduras (Gracias a Dios, El Paraíso, Comayagua, Atlántida, and Colón).

The majority (73%) of the anophelines were collected in the department of Gracias a Dios, which comprises a region known as La Moskitia, shared with Nicaragua, and endemic to malaria. La Moskitia exhibits unique ecological and socio-cultural characteristics. This region currently contributes 98% of malaria cases—195 out of 199 cases reported until epidemiological week number 5 of 2022 (Personal communication, Honduras Ministry of Health). The humid tropical ecosystem with abundant lagoons, as well as the scarce urban development of the region and a megadiverse fauna, could explain the great abundance of anophelines collected in the area.

In total, most of the specimens (80%) were identified as *An. albimanus*. This species was present in the five departments despite the ecological differences among the study sites. *Anopheles albimanus* has been described as the dominant species in most regions of Mesoamerica and northern South America [7]. *Anopheles albimanus* is classified as a generalist species, with the ability to inhabit a wide spectrum of ecosystems and to feed opportunistically on multiple hosts [14]. This finding is also consistent with a previous study conducted in Honduras, where 74% of anophelines identified were *An. albimanus* [3]. A study where 22,000 larvae of 13 species of anophelines were collected in 19 states of Mexico revealed that *An. albimanus* and *An. pseudopunctipennis* were the two most abundant species [31]. These two species were also found to be the most abundant and widely distributed anophelines along the Pacific coast of Mexico [32]. Two recent studies conducted among indigenous communities in Panama identified between 43% and 98% of mosquitoes as *An. albimanus* [33,34]. In Colombia, a retrospective descriptive study showed that *An. albimanus*, *An. nuneztovari s.l*., and *An. darlingi* were the main vectors in receptive areas for malaria [35], and a study assessing the potential distribution of the three main malaria vectors in Colombia determined that *An. albimanus* had the greatest niche breadth mainly in coastal areas [36].

The second species reported in this study and considered a dominant vector of malaria [7] was *An. pseudopunctipennis*, which was recorded only in Comayagua. *Anopheles pseudopunctipennis* appears to be a minority vector species in Honduras. Escobar et al. reported 3.1% of mosquitoes in Colón and Comayagua [3], while in this study, they amounted to less than 1%. The biotope that this species occupies likely has characteristics that limit its geographical distribution, so collections should be directed to those areas to increase the probability of capture. The remaining 17.4% of the collected mosquitoes belong to the following six species: *An. crucians*, *An. argyritarsis*, *An. neomaculipalpus*, *An. vestitipennis*, *An. apicimacula,* and *An. punctimacula*. Three of these species were not described in the 2020 study (*An. argyritarsis*, *An. apicimacula*, *An. neomaculipalpus)* [3], and are not considered dominant malaria vectors [6,37]. A notable difference from the 2020 study is the decrease in the proportion of two species collected in Gracias a Dios. The number of *Anopheles vestitipennis* decreased from 49.4% to 4.7%, and *An. crucians* decreased from 29.5% to 6.2% [3]. However, the differences in the relative abundance of these species could be influenced by the season of the year in which the mosquitoes were collected and the specific collection sites. The low proportions of uncommon *Anopheles* species found in this study suggest that malaria control in Honduras should continue to focus on the most abundant *An. albimanus* and *An. pseudopunctipennis*. No *An. darlingi* or *An. neivai* specimens were collected in this study, which could be related to the limited geographical distribution of these species in Honduras, having been reported in the department of Atlántida [3].

*Anopheles argyritarsis* was collected in Puerto Lempira, Gracias a Dios, and in San José de Comayagua. Although this species is widely distributed in the Neotropics [38,39,40,41], its potential as a malaria vector is controversial, with evidence for insufficient or non-existent vectorial capacity [40,42]. *Anopheles apicimacula* and *An. neomaculipalpus* were collected only in Gracias a Dios. Both species have widely been reported in Mesoamerica [43,44,45] and South America [46,47,48,49,50,51,52,53,54,55], and have historically been reported by the Honduran health authorities (unpublished data), but natural infections with *Plasmodium* sporozoites have only been detected in *An. neomaculipalpus* [55].

In addition to taxonomic identification, a partial segment of the *COI* gene was amplified and sequenced to confirm the identity of some specimens and decipher intraspecies genetic variability. All morphologically identified individuals were molecularly confirmed by barcoding. These results confirm the usefulness of the mitochondrial genome as a genetic marker [4,21]. In this study, the molecular identification of specimens collected in Roatán (Bay Islands), located more than 68 km from the mainland, was performed for the first time. All mosquitoes collected on the island were identified as *An. albimanus* and the phylogenetic analyses revealed no geographical-region-based clustering, suggesting genetic flow between both populations. This result supports those of Molina-Cruz et al. [56], after analysing a large population of mosquitoes from the Caribbean, Central America, and South America, using microsatellites. These authors demonstrated little genetic variation among the populations of northern Central America and weak isolation by distance. However, it has been suggested that there might be some barrier to gene flow [56] or contemporary isolation by distance in the isthmus [57] between the populations of *An. albimanus* from northern Central America and those of Panama and South America.

Herein, we reported the first *COI* sequences for *An. argyritarsis*, *An. neomaculipalpus*, and *An. apicimacula* from Honduras. When comparing the sequences obtained here with homologous sequences of *An. argyritarsis*, a clear separation was found between the populations of Brazil/Colombia and the populations of Honduras/Mexico. A similar pattern was observed in the *An. neomaculipalpus* cladogram, in which the sequences from Colombia and Honduras are separated. Although the number of sequences is small, it is possible to speculate that there is geographical isolation between the Central American and South American populations, perhaps imposed by both the geographical distance and the so-called Darién Gap between Panama and Colombia. Further analyses including a greater number of individuals and the use of more robust molecular markers such as microsatellites could help decipher the evolutionary relationship of this species in the Neotropics. For *An. apicimacula*, no geographical separation was observed between the sequences from Honduras and those from Colombia even though the low percentage of identity yielded via the BLAST tool (93.5%) with respect to the sequences previously deposited in GenBank. According to the ‘barcoding gap’ hypothesis, pairwise genetic differences greater than 3% are suggestive of separation between two species. Recent studies have described two geographically isolated lineages of *An. apicimacula* in Colombia [46,48], which supports the existence of an Apicimacula species complex that would encompass several species, including the specimens from Honduras. This question should be studied further in the future to clarify the taxonomy of this species.

Finally, none of the tested mosquitoes were positive for *Plasmodium* DNA using conventional *mt cox1* gene PCR, which has been proven to be more sensitive than the CSP antigen detection by using ELISA [58]. Several authors have reported mosquitoes infected by *Plasmodium* spp. in Africa [59,60,61,62] and the Amazon Basin in South America [47,51,55] where higher rates of transmission have been reported. The absence of infected mosquitoes was not unexpected due to the low number of malaria cases reported in Gracias a Dios, Bay Islands, and El Paraíso, during the years of collection (average of 380, 7, and 18 cases per year, respectively), and the absence of malaria cases in Cortés and Comayagua. This result suggests that a greater number of samples will be necessary to find infected mosquitoes given the current epidemiological situation in the country. The main limitation of this follow-up study is the low number of mosquitoes collected, despite multiple visits to five departments of the country over three years. The poor return on investment in sampling efforts may be attributed to the season of the year in which the collections were made or to other factors that are not fully understood. Future studies should take these results into account to increase the sample size.

## 5. Conclusions

This study revises the distribution, diversity, and abundance of anopheline populations in Honduras, revealing a notable predominance of *An. albimanus*. This is the first report of COI gene sequences for three anopheline species collected in Honduras (*An. argyritarsis*, *An. apicimacula*, and *An. neomaculipalpus*). Our results suggest that geographical isolation is possible between *An. argyritarsis* and *An. neomaculipalpus* populations in Central and South America. This is also the first report of COI sequences of *An. albimanus* collected on the island of Roatán, Bay Islands, with apparent gene flow relative to mainland populations.

## Figures and Tables

**Table 1:** Collection sites’ coordinates, altitude, date of collection, and number of *Anopheles* mosquitoes collected.

Department	Municipality	Coordinates (Latitude and Longitude)	Altitude (masl)	Number of Specimens Collected	Date of Collection
Gracias a Dios	Puerto Lempira	14.93567985, −83.84507528/14.94412734, −83.82883771/15.25098087, −83.77352977/15.31331402, −83.5747129/14.700630, −84.335100	7–35	211	July to September 2021
Bay Islands	Roatán	16.323647, −86.563377	62	32	September 2021
Comayagua	Comayagua	14.650778, −87.608472/14.627806, −87.605806/14.650778, −87.608472/14.651083, −87.609444/14.650333, −87.607472	430–640	23	June 2018 to May 2021
Comayagua	La Libertad	14.759750, −87.614861	392		March 2019
Comayagua	San José	14.735295, −88.029439	701		July 2021
El Paraíso	Morocelí	14.103917, −86.918417/14.104639, −86.919111/14.102944, −86.917694	605	19	August 2019
Cortés	Pimienta	15.289617, −88.029439/15.289617, −87.977116	47–237	3	August 2021
Cortés	Santa Cruz de Yojoa	14.855360, −87.929549	742		June 2021

**Table 2:** Distribution and abundance of *Anopheles* species by geographical region.

Department	Municipality	*An.* (*Nyssorhyncus*) *albimanus*	*An.* (*Nyssorhyncus*) *argyritarsis*	*An.* (*Anopheles*) *pseudopunctipennis*	*An.* (*Anopheles*) *apicimacula*	*An.* (*Anopheles*) *neomaculipalpus*	*An.* (*Anopheles*) *punctimacula*	*An.* (*Anopheles*) *crucians*	*An.* (*Anopheles*) *vestitipennis*	Total (%)
Gracias a Dios	Puerto Lempira	168	4	-	3	11	2	13	10	211 (73.3%)
Bay Islands	Roatán	32	-	-	-	-	-	-	-	32 (11.1%)
Comayagua	Comayagua	7	-	7	-	-	-	-	-	14 (4.9%)
Comayagua	La Libertad	-	-	2	-	-	-	-	-	2 (0.69%)
Comayagua	San José	-	7	-	-	-	-	-	-	7 (2.4%)
El Paraíso	Morocelí	19	-	-	-	-	-	-	-	19 (6.6%)
Cortés	Pimienta	2	-	-	-	-	-	-	-	2 (0.69%)
Cortés	Santa Cruz de Yojoa	1	-	-	-	-	-	-	-	1 (0.35%)
Total (%)		229 (79.51%)	11 (3.82%)	9 (3.13%)	3 (1.04%)	11 (3.82%)	2 (0.69%)	13 (4.51%)	10 (3.47%)	288 (100%)

**Table 3:** Intraspecific comparison of nucleotide sequences, genetic diversity, and, number of haplotypes for *COI* in 8 species of *Anopheles* from Honduras.

Feature	*An.* (*Nys.*) *albimanus*	*An.* (*Nys.*) *argyritarsis*	*An.* (*An.*) *pseudopunctipennis*	*An.* (*An.*) *apicimacula*	*An.* (*An.*) *neomaculipalpus*	*An.* (*An.*) *punctimacula*	*An.* (*An.*) *crucians*	*An.* (*An.*) *vestitipennis*
Nucleotide sequence length	581	596	598	652	572	530	611	615
Number of sequences analysed	89	16	14	7	21	2	20	6
Identical sites	544	589	583	612	567	-	584	607
Identical sites (%)	93.6%	98.8%	97.5%	94.0%	99.1%	-	95.6%	98.9%
Pairwise % identity	99.1%	99.7%	99.3%	96.0%	99.7%	-	98.2%	99.4%
π	0.01	0.01	0.01	0.04	0.00	-	0.02	0.01
Haplotype								
Number of sequences analysed	80	10	8	3	11	2	8	5
Nº of haplotypes (nucleotide)	35	6	5	3	4	-	8	3
Haplotypes/N	0.44	0.6	0.62	1	0.4	-	1	0.6
Haplotype diversity	0.94	0.84	0.79	1	0.8	-	1	0.7
Aminoacid sequence length	209	198	198	216	190	176	203	204
Nº of haplotypes (amino acid)	5	2	1	1	1	-	1	1

## Data Availability

Data supporting the conclusions of this article are included within the article and its additional files. The raw data used and/or analysed during the present study are available from the corresponding author on reasonable request.

## Supplementary Materials

The following supporting information can be downloaded at: https://www.mdpi.com/article/10.3390/insects13060548/s1, Table S1. Description of the anopheline species found by municipality and result of the DNA detection analysis of *Plasmodium* spp.

## Copyright Statement

Some authors of this manuscript are military service members and employees of the US Government. This work was prepared as part of their official duties. Title 17 USC §105 provides that copyright protection under this title is not available for any work of the United States Government. Title 17 USC §101 defines a US Government work as work prepared by a military service member or employee of the US Government as part of that person’s official duties.
