# Supplementary material for: A Follow-Up to the Geographical Distribution of Anopheles Species in Malaria-Endemic and Non-Endemic Areas of Honduras
Source: Insects. 2022 Jun 15;13(6):548. doi: 10.3390/insects13060548 (PMC9225189; doi:10.3390/insects13060548)
Supplement: Supplementary file 1 [file insects-13-00548-s001.zip › insects-1734820-supplementary.pdf]

Table S1. Description of the anopheline species found by municipality and result of the DNA detection analysis of *Plasmodium* spp.

No	COD	Department	Municipality	Locality	Collection Method	Family	*Genus*	Subgenus	Species	Plasmodium_cox1
1	HND-2021-GAD-015-0001	Gracias a Dios	Puerto Lempira	Lisagnia	CDC Light Trap	Culicidae	*Anopheles*	*Nyssorhyncus*	*argyritarsis*	NEG
2	HND-2021-GAD-016-0003	Gracias a Dios	Puerto Lempira	Yabaltara	CDC Light Trap	Culicidae	*Anopheles*	*Nyssorhyncus*	*albimanus*	Not processed
3	HND-2021-GAD-016-0004	Gracias a Dios	Puerto Lempira	Yabaltara	CDC Light Trap	Culicidae	*Anopheles*	*Nyssorhyncus*	*albimanus*	Not processed
4	HND-2021-GAD-016-0005	Gracias a Dios	Puerto Lempira	Yabaltara	CDC Light Trap	Culicidae	*Anopheles*	*Nyssorhyncus*	*albimanus*	Not processed
5	HND-2021-GAD-022-0011	Gracias a Dios	Puerto Lempira	Auka centro	Resting collection	Culicidae	*Anopheles*	*Nyssorhyncus*	*albimanus*	NEG
6	HND-2021-GAD-022-0014	Gracias a Dios	Puerto Lempira	Auka centro	Resting collection	Culicidae	*Anopheles*	*Nyssorhyncus*	*albimanus*	Not processed
7	HND-2021-GAD-022-0015	Gracias a Dios	Puerto Lempira	Auka centro	Resting collection	Culicidae	*Anopheles*	*Nyssorhyncus*	*albimanus*	Not processed
8	HND-2021-GAD-022-0032	Gracias a Dios	Puerto Lempira	Auka centro	Resting collection	Culicidae	*Anopheles*	*Nyssorhyncus*	*albimanus*	Not processed
9	HND-2021-GAD-022-0033	Gracias a Dios	Puerto Lempira	Auka centro	Resting collection	Culicidae	*Anopheles*	*Nyssorhyncus*	*albimanus*	Not processed
10	HND-2021-GAD-022-0034	Gracias a Dios	Puerto Lempira	Auka centro	Resting collection	Culicidae	*Anopheles*	*Nyssorhyncus*	*albimanus*	Not processed
11	HND-2021-GAD-022-0038	Gracias a Dios	Puerto Lempira	Auka centro	Resting collection	Culicidae	*Anopheles*	*Nyssorhyncus*	*albimanus*	NEG
12	HND-2021-GAD-022-0039	Gracias a Dios	Puerto Lempira	Auka centro	Resting collection	Culicidae	*Anopheles*	*Nyssorhyncus*	*albimanus*	Not processed
13	HND-2021-GAD-022-0040	Gracias a Dios	Puerto Lempira	Auka centro	Resting collection	Culicidae	*Anopheles*	*Nyssorhyncus*	*albimanus*	Not processed
14	HND-2021-GAD-022-0043	Gracias a Dios	Puerto Lempira	Auka centro	Resting collection	Culicidae	*Anopheles*	*Nyssorhyncus*	*albimanus*	NEG
15	HND-2021-GAD-022-0044	Gracias a Dios	Puerto Lempira	Auka centro	Resting collection	Culicidae	*Anopheles*	*Nyssorhyncus*	*albimanus*	NEG
16	HND-2021-GAD-014-0049	Gracias a Dios	Puerto Lempira	Barrio Alabar	Resting collection	Culicidae	*Anopheles*	*Nyssorhyncus*	*albimanus*	Not processed
17	HND-2021-GAD-022-0050	Gracias a Dios	Puerto Lempira	Auka centro	Resting collection	Culicidae	*Anopheles*	*Nyssorhyncus*	*albimanus*	Not processed
18	HND-2021-GAD-022-0051	Gracias a Dios	Puerto Lempira	Auka centro	Resting collection	Culicidae	*Anopheles*	*Nyssorhyncus*	*albimanus*	Not processed
19	HND-2021-GAD-022-0052	Gracias a Dios	Puerto Lempira	Auka centro	Resting collection	Culicidae	*Anopheles*	*Nyssorhyncus*	*albimanus*	Not processed
20	HND-2021-GAD-022-0053	Gracias a Dios	Puerto Lempira	Auka centro	Resting collection	Culicidae	*Anopheles*	*Nyssorhyncus*	*albimanus*	Not processed
21	HND-2021-GAD-022-0054	Gracias a Dios	Puerto Lempira	Auka centro	Resting collection	Culicidae	*Anopheles*	*Nyssorhyncus*	*albimanus*	Not processed
22	HND-2021-GAD-022-0055	Gracias a Dios	Puerto Lempira	Auka centro	Resting collection	Culicidae	*Anopheles*	*Nyssorhyncus*	*albimanus*	Not processed
23	HND-2021-GAD-022-0056	Gracias a Dios	Puerto Lempira	Auka centro	Resting collection	Culicidae	*Anopheles*	*Nyssorhyncus*	*albimanus*	Not processed

24	HND-2021-GAD-022-0059	Gracias a Dios	Puerto Lempira	Auka centro	Resting collection	Culicidae	*Anopheles*	*Nyssorhyncus*	*albimanus*	Not processed
25	HND-2021-GAD-022-0060	Gracias a Dios	Puerto Lempira	Auka centro	Resting collection	Culicidae	*Anopheles*	*Nyssorhyncus*	*albimanus*	Not processed
26	HND-2021-GAD-022-0061	Gracias a Dios	Puerto Lempira	Auka centro	Resting collection	Culicidae	*Anopheles*	*Nyssorhyncus*	*albimanus*	Not processed
27	HND-2021-GAD-022-0063	Gracias a Dios	Puerto Lempira	Auka centro	Resting collection	Culicidae	*Anopheles*	*Nyssorhyncus*	*albimanus*	Not processed
28	HND-2021-GAD-022-0064	Gracias a Dios	Puerto Lempira	Auka centro	Resting collection	Culicidae	*Anopheles*	*Nyssorhyncus*	*albimanus*	Not processed
29	HND-2021-GAD-022-0065	Gracias a Dios	Puerto Lempira	Auka centro	Resting collection	Culicidae	*Anopheles*	*Nyssorhyncus*	*albimanus*	Not processed
30	HND-2021-GAD-022-0066	Gracias a Dios	Puerto Lempira	Auka centro	Resting collection	Culicidae	*Anopheles*	*Nyssorhyncus*	*albimanus*	Not processed
31	HND-2021-GAD-022-0067	Gracias a Dios	Puerto Lempira	Auka centro	Resting collection	Culicidae	*Anopheles*	*Nyssorhyncus*	*albimanus*	Not processed
32	HND-2021-GAD-022-0068	Gracias a Dios	Puerto Lempira	Auka centro	Resting collection	Culicidae	*Anopheles*	*Nyssorhyncus*	*albimanus*	NEG
33	HND-2021-GAD-001-0069	Gracias a Dios	Puerto Lempira	Barrio Usupum	Shannon trap	Culicidae	*Anopheles*	*Anopheles*	*crucians*	NEG
34	HND-2021-GAD-022-0070	Gracias a Dios	Puerto Lempira	Auka centro	Resting collection	Culicidae	*Anopheles*	*Nyssorhyncus*	*albimanus*	NEG
35	HND-2021-GAD-022-0071	Gracias a Dios	Puerto Lempira	Auka centro	Resting collection	Culicidae	*Anopheles*	*Nyssorhyncus*	*albimanus*	Not processed
36	HND-2021-GAD-022-0072	Gracias a Dios	Puerto Lempira	Auka centro	Resting collection	Culicidae	*Anopheles*	*Nyssorhyncus*	*albimanus*	Not processed
37	HND-2021-GAD-022-0073	Gracias a Dios	Puerto Lempira	Auka centro	Resting collection	Culicidae	*Anopheles*	*Nyssorhyncus*	*albimanus*	Not processed
38	HND-2021-GAD-022-0074	Gracias a Dios	Puerto Lempira	Auka centro	Resting collection	Culicidae	*Anopheles*	*Nyssorhyncus*	*albimanus*	NEG
39	HND-2021-GAD-022-0075	Gracias a Dios	Puerto Lempira	Auka centro	Resting collection	Culicidae	*Anopheles*	*Nyssorhyncus*	*albimanus*	NEG
40	HND-2021-GAD-005-0076	Gracias a Dios	Puerto Lempira	Dapat	CDC Light Trap	Culicidae	*Anopheles*	*Nyssorhyncus*	*albimanus*	NEG
41	HND-2021-GAD-022-0077	Gracias a Dios	Puerto Lempira	Auka centro	Resting collection	Culicidae	*Anopheles*	*Nyssorhyncus*	*albimanus*	Not processed
42	HND-2021-GAD-022-0078	Gracias a Dios	Puerto Lempira	Auka centro	Resting collection	Culicidae	*Anopheles*	*Nyssorhyncus*	*albimanus*	Not processed
43	HND-2021-GAD-022-0079	Gracias a Dios	Puerto Lempira	Auka centro	Resting collection	Culicidae	*Anopheles*	*Nyssorhyncus*	*albimanus*	NEG
44	HND-2021-GAD-022-0080	Gracias a Dios	Puerto Lempira	Auka centro	Resting collection	Culicidae	*Anopheles*	*Nyssorhyncus*	*albimanus*	NEG
45	HND-2021-GAD-022-0081	Gracias a Dios	Puerto Lempira	Auka centro	Resting collection	Culicidae	*Anopheles*	*Nyssorhyncus*	*albimanus*	Not processed
46	HND-2021-GAD-022-0082	Gracias a Dios	Puerto Lempira	Auka centro	Resting collection	Culicidae	*Anopheles*	*Nyssorhyncus*	*albimanus*	Not processed
47	HND-2021-GAD-022-0092	Gracias a Dios	Puerto Lempira	Auka centro	Resting collection	Culicidae	*Anopheles*	*Nyssorhyncus*	*albimanus*	Not processed
48	HND-2021-GAD-022-0093	Gracias a Dios	Puerto Lempira	Auka centro	Resting collection	Culicidae	*Anopheles*	*Nyssorhyncus*	*albimanus*	Not processed
49	HND-2021-GAD-022-0094	Gracias a Dios	Puerto Lempira	Auka centro	Resting collection	Culicidae	*Anopheles*	*Nyssorhyncus*	*albimanus*	NEG
50	HND-2021-GAD-022-0098	Gracias a Dios	Puerto Lempira	Auka centro	Resting collection	Culicidae	*Anopheles*	*Nyssorhyncus*	*albimanus*	NEG

51	HND-2021-GAD-022-0099	Gracias a Dios	Puerto Lempira	Auka centro	Resting collection	Culicidae	*Anopheles*	*Nyssorhyncus*	*albimanus*	NEG
52	HND-2021-GAD-022-0100	Gracias a Dios	Puerto Lempira	Auka centro	Resting collection	Culicidae	*Anopheles*	*Nyssorhyncus*	*albimanus*	NEG
53	HND-2021-GAD-022-0101	Gracias a Dios	Puerto Lempira	Auka centro	Resting collection	Culicidae	*Anopheles*	*Nyssorhyncus*	*albimanus*	NEG
54	HND-2021-GAD-022-0102	Gracias a Dios	Puerto Lempira	Auka centro	Resting collection	Culicidae	*Anopheles*	*Nyssorhyncus*	*albimanus*	NEG
55	HND-2021-GAD-022-0103	Gracias a Dios	Puerto Lempira	Auka centro	Resting collection	Culicidae	*Anopheles*	*Nyssorhyncus*	*albimanus*	NEG
56	HND-2021-GAD-022-0107	Gracias a Dios	Puerto Lempira	Auka centro	Resting collection	Culicidae	*Anopheles*	*Nyssorhyncus*	*albimanus*	NEG
57	HND-2021-GAD-022-0108	Gracias a Dios	Puerto Lempira	Auka centro	Resting collection	Culicidae	*Anopheles*	*Nyssorhyncus*	*albimanus*	Not processed
58	HND-2021-GAD-022-0109	Gracias a Dios	Puerto Lempira	Auka centro	Resting collection	Culicidae	*Anopheles*	*Nyssorhyncus*	*albimanus*	Not processed
59	HND-2021-GAD-022-0110	Gracias a Dios	Puerto Lempira	Auka centro	Resting collection	Culicidae	*Anopheles*	*Nyssorhyncus*	*albimanus*	NEG
60	HND-2021-GAD-022-0111	Gracias a Dios	Puerto Lempira	Auka centro	Resting collection	Culicidae	*Anopheles*	*Nyssorhyncus*	*albimanus*	NEG
61	HND-2021-GAD-022-0112	Gracias a Dios	Puerto Lempira	Auka centro	Resting collection	Culicidae	*Anopheles*	*Nyssorhyncus*	*albimanus*	NEG
62	HND-2021-GAD-022-0115	Gracias a Dios	Puerto Lempira	Auka centro	Resting collection	Culicidae	*Anopheles*	*Nyssorhyncus*	*albimanus*	Not processed
63	HND-2021-GAD-022-0116	Gracias a Dios	Puerto Lempira	Auka centro	Resting collection	Culicidae	*Anopheles*	*Nyssorhyncus*	*albimanus*	NEG
64	HND-2021-GAD-022-0117	Gracias a Dios	Puerto Lempira	Auka centro	Resting collection	Culicidae	*Anopheles*	*Nyssorhyncus*	*albimanus*	NEG
65	HND-2021-GAD-022-0118	Gracias a Dios	Puerto Lempira	Auka centro	Resting collection	Culicidae	*Anopheles*	*Nyssorhyncus*	*albimanus*	NEG
66	HND-2021-GAD-022-0119	Gracias a Dios	Puerto Lempira	Auka centro	Resting collection	Culicidae	*Anopheles*	*Nyssorhyncus*	*albimanus*	NEG
67	HND-2021-GAD-022-0120	Gracias a Dios	Puerto Lempira	Auka centro	Resting collection	Culicidae	*Anopheles*	*Nyssorhyncus*	*albimanus*	NEG
68	HND-2021-GAD-022-0121	Gracias a Dios	Puerto Lempira	Auka centro	Resting collection	Culicidae	*Anopheles*	*Nyssorhyncus*	*albimanus*	NEG
69	HND-2021-GAD-022-0122	Gracias a Dios	Puerto Lempira	Auka centro	Resting collection	Culicidae	*Anopheles*	*Nyssorhyncus*	*albimanus*	NEG
70	HND-2021-GAD-022-0123	Gracias a Dios	Puerto Lempira	Auka centro	Resting collection	Culicidae	*Anopheles*	*Nyssorhyncus*	*albimanus*	NEG
71	HND-2021-GAD-022-0127	Gracias a Dios	Puerto Lempira	Auka centro	Resting collection	Culicidae	*Anopheles*	*Nyssorhyncus*	*albimanus*	NEG
72	HND-2021-GAD-022-0128	Gracias a Dios	Puerto Lempira	Auka centro	Resting collection	Culicidae	*Anopheles*	*Nyssorhyncus*	*albimanus*	NEG
73	HND-2021-GAD-022-0129	Gracias a Dios	Puerto Lempira	Auka centro	Resting collection	Culicidae	*Anopheles*	*Nyssorhyncus*	*albimanus*	NEG
74	HND-2021-GAD-022-0130	Gracias a Dios	Puerto Lempira	Auka centro	Resting collection	Culicidae	*Anopheles*	*Nyssorhyncus*	*albimanus*	NEG
75	HND-2021-GAD-022-0131	Gracias a Dios	Puerto Lempira	Auka centro	Resting collection	Culicidae	*Anopheles*	*Nyssorhyncus*	*albimanus*	NEG
76	HND-2021-GAD-022-0132	Gracias a Dios	Puerto Lempira	Auka centro	Resting collection	Culicidae	*Anopheles*	*Nyssorhyncus*	*albimanus*	NEG
77	HND-2021-GAD-022-0133	Gracias a Dios	Puerto Lempira	Auka centro	Resting collection	Culicidae	*Anopheles*	*Nyssorhyncus*	*albimanus*	NEG

78	HND-2021-GAD-022-0134	Gracias a Dios	Puerto Lempira	Auka centro	Resting collection	Culicidae	*Anopheles*	*Nyssorhyncus*	*albimanus*	NEG
79	HND-2021-GAD-022-0135	Gracias a Dios	Puerto Lempira	Auka centro	Resting collection	Culicidae	*Anopheles*	*Nyssorhyncus*	*albimanus*	NEG
80	HND-2021-GAD-022-0136	Gracias a Dios	Puerto Lempira	Auka centro	Resting collection	Culicidae	*Anopheles*	*Nyssorhyncus*	*albimanus*	NEG
81	HND-2021-GAD-022-0137	Gracias a Dios	Puerto Lempira	Auka centro	Resting collection	Culicidae	*Anopheles*	*Nyssorhyncus*	*albimanus*	NEG
82	HND-2021-GAD-012-0138	Gracias a Dios	Puerto Lempira	Cocodacra	Resting collection	Culicidae	*Anopheles*	*Nyssorhyncus*	*albimanus*	NEG
83	HND-2021-GAD-022-0139	Gracias a Dios	Puerto Lempira	Auka centro	Resting collection	Culicidae	*Anopheles*	*Anopheles*	*vestitipennis*	Not processed
84	HND-2021-GAD-022-0140	Gracias a Dios	Puerto Lempira	Auka centro	Resting collection	Culicidae	*Anopheles*	*Anopheles*	*vestitipennis*	Not processed
85	HND-2021-GAD-022-0141	Gracias a Dios	Puerto Lempira	Auka centro	Resting collection	Culicidae	*Anopheles*	*Anopheles*	*vestitipennis*	Not processed
86	HND-2021-GAD-023-0142	Gracias a Dios	Puerto Lempira	Suhi	Resting collection	Culicidae	*Anopheles*	*Nyssorhyncus*	*albimanus*	Not processed
87	HND-2021-GAD-023-0143	Gracias a Dios	Puerto Lempira	Suhi	Resting collection	Culicidae	*Anopheles*	*Nyssorhyncus*	*albimanus*	Not processed
88	HND-2021-GAD-023-0144	Gracias a Dios	Puerto Lempira	Suhi	Resting collection	Culicidae	*Anopheles*	*Nyssorhyncus*	*albimanus*	Not processed
89	HND-2021-GAD-023-0145	Gracias a Dios	Puerto Lempira	Suhi	Resting collection	Culicidae	*Anopheles*	*Nyssorhyncus*	*albimanus*	Not processed
90	HND-2021-GAD-023-0146	Gracias a Dios	Puerto Lempira	Suhi	Resting collection	Culicidae	*Anopheles*	*Nyssorhyncus*	*albimanus*	Not processed
91	HND-2021-GAD-023-0147	Gracias a Dios	Puerto Lempira	Suhi	Resting collection	Culicidae	*Anopheles*	*Nyssorhyncus*	*albimanus*	Not processed
92	HND-2021-GAD-023-0148	Gracias a Dios	Puerto Lempira	Suhi	Resting collection	Culicidae	*Anopheles*	*Nyssorhyncus*	*albimanus*	NEG
93	HND-2021-GAD-023-0149	Gracias a Dios	Puerto Lempira	Suhi	Resting collection	Culicidae	*Anopheles*	*Nyssorhyncus*	*albimanus*	NEG
94	HND-2021-GAD-023-0150	Gracias a Dios	Puerto Lempira	Suhi	Resting collection	Culicidae	*Anopheles*	*Nyssorhyncus*	*albimanus*	NEG
95	HND-2021-GAD-023-0151	Gracias a Dios	Puerto Lempira	Suhi	Resting collection	Culicidae	*Anopheles*	*Nyssorhyncus*	*albimanus*	Not processed
96	HND-2021-GAD-023-0152	Gracias a Dios	Puerto Lempira	Suhi	Resting collection	Culicidae	*Anopheles*	*Nyssorhyncus*	*albimanus*	Not processed
97	HND-2021-GAD-023-0153	Gracias a Dios	Puerto Lempira	Suhi	Resting collection	Culicidae	*Anopheles*	*Nyssorhyncus*	*albimanus*	Not processed
98	HND-2021-GAD-023-0154	Gracias a Dios	Puerto Lempira	Suhi	Resting collection	Culicidae	*Anopheles*	*Nyssorhyncus*	*albimanus*	NEG
99	HND-2021-GAD-023-0155	Gracias a Dios	Puerto Lempira	Suhi	Resting collection	Culicidae	*Anopheles*	*Nyssorhyncus*	*albimanus*	NEG
100	HND-2021-GAD-023-0156	Gracias a Dios	Puerto Lempira	Suhi	Resting collection	Culicidae	*Anopheles*	*Nyssorhyncus*	*albimanus*	NEG
101	HND-2021-GAD-023-0157	Gracias a Dios	Puerto Lempira	Suhi	Resting collection	Culicidae	*Anopheles*	*Nyssorhyncus*	*albimanus*	NEG
102	HND-2021-GAD-023-0158	Gracias a Dios	Puerto Lempira	Suhi	Resting collection	Culicidae	*Anopheles*	*Nyssorhyncus*	*albimanus*	NEG
103	HND-2021-GAD-023-0159	Gracias a Dios	Puerto Lempira	Suhi	Resting collection	Culicidae	*Anopheles*	*Nyssorhyncus*	*albimanus*	NEG
104	HND-2021-GAD-023-0160	Gracias a Dios	Puerto Lempira	Suhi	Resting collection	Culicidae	*Anopheles*	*Nyssorhyncus*	*albimanus*	Not processed

105	HND-2021-GAD-023-0161	Gracias a Dios	Puerto Lempira	Suhi	Resting collection	Culicidae	*Anopheles*	*Nyssorhyncus*	*albimanus*	Not processed
106	HND-2021-GAD-023-0162	Gracias a Dios	Puerto Lempira	Suhi	Resting collection	Culicidae	*Anopheles*	*Nyssorhyncus*	*albimanus*	Not processed
107	HND-2021-GAD-023-0163	Gracias a Dios	Puerto Lempira	Suhi	Resting collection	Culicidae	*Anopheles*	*Nyssorhyncus*	*albimanus*	NEG
108	HND-2021-GAD-023-0164	Gracias a Dios	Puerto Lempira	Suhi	Resting collection	Culicidae	*Anopheles*	*Nyssorhyncus*	*albimanus*	NEG
109	HND-2021-GAD-023-0165	Gracias a Dios	Puerto Lempira	Suhi	Resting collection	Culicidae	*Anopheles*	*Nyssorhyncus*	*albimanus*	NEG
110	HND-2021-GAD-023-0166	Gracias a Dios	Puerto Lempira	Suhi	Resting collection	Culicidae	*Anopheles*	*Nyssorhyncus*	*albimanus*	NEG
111	HND-2021-GAD-023-0167	Gracias a Dios	Puerto Lempira	Suhi	Resting collection	Culicidae	*Anopheles*	*Nyssorhyncus*	*albimanus*	NEG
112	HND-2021-GAD-023-0168	Gracias a Dios	Puerto Lempira	Suhi	Resting collection	Culicidae	*Anopheles*	*Nyssorhyncus*	*albimanus*	NEG
113	HND-2021-GAD-023-0169	Gracias a Dios	Puerto Lempira	Suhi	Resting collection	Culicidae	*Anopheles*	*Nyssorhyncus*	*albimanus*	Not processed
114	HND-2021-GAD-023-0170	Gracias a Dios	Puerto Lempira	Suhi	Resting collection	Culicidae	*Anopheles*	*Nyssorhyncus*	*albimanus*	Not processed
115	HND-2021-GAD-023-0171	Gracias a Dios	Puerto Lempira	Suhi	Resting collection	Culicidae	*Anopheles*	*Nyssorhyncus*	*albimanus*	NEG
116	HND-2021-GAD-023-0172	Gracias a Dios	Puerto Lempira	Suhi	Resting collection	Culicidae	*Anopheles*	*Nyssorhyncus*	*albimanus*	NEG
117	HND-2021-GAD-023-0173	Gracias a Dios	Puerto Lempira	Suhi	Resting collection	Culicidae	*Anopheles*	*Nyssorhyncus*	*albimanus*	NEG
118	HND-2021-GAD-023-0174	Gracias a Dios	Puerto Lempira	Suhi	Resting collection	Culicidae	*Anopheles*	*Nyssorhyncus*	*albimanus*	NEG
119	HND-2021-GAD-023-0175	Gracias a Dios	Puerto Lempira	Suhi	Resting collection	Culicidae	*Anopheles*	*Nyssorhyncus*	*albimanus*	NEG
120	HND-2021-GAD-023-0176	Gracias a Dios	Puerto Lempira	Suhi	Resting collection	Culicidae	*Anopheles*	*Nyssorhyncus*	*albimanus*	NEG
121	HND-2021-GAD-023-0177	Gracias a Dios	Puerto Lempira	Suhi	Resting collection	Culicidae	*Anopheles*	*Anopheles*	*vestitipennis*	NEG
122	HND-2021-GAD-023-0178	Gracias a Dios	Puerto Lempira	Suhi	Resting collection	Culicidae	*Anopheles*	*Anopheles*	*vestitipennis*	NEG
123	HND-2021-GAD-023-0179	Gracias a Dios	Puerto Lempira	Suhi	Resting collection	Culicidae	*Anopheles*	*Anopheles*	*vestitipennis*	NEG
124	HND-2021-GAD-023-0180	Gracias a Dios	Puerto Lempira	Suhi	Resting collection	Culicidae	*Anopheles*	*Anopheles*	*vestitipennis*	Not processed
125	HND-2021-GAD-023-0181	Gracias a Dios	Puerto Lempira	Suhi	Resting collection	Culicidae	*Anopheles*	*Anopheles*	*vestitipennis*	Not processed
126	HND-2021-GAD-023-0182	Gracias a Dios	Puerto Lempira	Suhi	Resting collection	Culicidae	*Anopheles*	*Anopheles*	*neomaculipalpus*	Not processed
127	HND-2021-GAD-023-0183	Gracias a Dios	Puerto Lempira	Suhi	Resting collection	Culicidae	*Anopheles*	*Anopheles*	*neomaculipalpus*	NEG
128	HND-2021-GAD-023-0184	Gracias a Dios	Puerto Lempira	Suhi	Resting collection	Culicidae	*Anopheles*	*Anopheles*	*neomaculipalpus*	NEG
129	HND-2021-GAD-023-0185	Gracias a Dios	Puerto Lempira	Suhi	Resting collection	Culicidae	*Anopheles*	*Anopheles*	*apicimacula*	NEG
130	HND-2021-GAD-023-0186	Gracias a Dios	Puerto Lempira	Suhi	Resting collection	Culicidae	*Anopheles*	*Nyssorhyncus*	*apicimacula*	NEG
131	HND-2021-GAD-023-0187	Gracias a Dios	Puerto Lempira	Suhi	Resting collection	Culicidae	*Anopheles*	*Anopheles*	*punctimacula*	NEG

132	HND-2021-GAD-023-0188	Gracias a Dios	Puerto Lempira	Suhi	Resting collection	Culicidae	*Anopheles*	*Anopheles*	*neomaculipalpus*	NEG
133	HND-2021-GAD-023-0189	Gracias a Dios	Puerto Lempira	Suhi	Resting collection	Culicidae	*Anopheles*	*Nyssorhyncus*	*apicimacula*	NEG
134	HND-2021-GAD-023-0190	Gracias a Dios	Puerto Lempira	Suhi	Resting collection	Culicidae	*Anopheles*	*Anopheles*	*vestitipennis*	NEG
135	HND-2021-GAD-023-0191	Gracias a Dios	Puerto Lempira	Suhi	Resting collection	Culicidae	*Anopheles*	*Anopheles*	*crucians*	NEG
136	HND-2021-GAD-023-0192	Gracias a Dios	Puerto Lempira	Suhi	Resting collection	Culicidae	*Anopheles*	*Anopheles*	*crucians*	NEG
137	HND-2021-GAD-023-0193	Gracias a Dios	Puerto Lempira	Suhi	Resting collection	Culicidae	*Anopheles*	*Nyssorhyncus*	*albimanus*	Not processed
138	HND-2021-GAD-023-0194	Gracias a Dios	Puerto Lempira	Suhi	Resting collection	Culicidae	*Anopheles*	*Anopheles*	*crucians*	NEG
139	HND-2021-GAD-023-0196	Gracias a Dios	Puerto Lempira	Suhi	Resting collection	Culicidae	*Anopheles*	*Anopheles*	*crucians*	Not processed
140	HND-2021-GAD-023-0197	Gracias a Dios	Puerto Lempira	Suhi	Resting collection	Culicidae	*Anopheles*	*Anopheles*	*crucians*	Not processed
141	HND-2021-GAD-023-0198	Gracias a Dios	Puerto Lempira	Suhi	Resting collection	Culicidae	*Anopheles*	*Anopheles*	*crucians*	Not processed
142	HND-2021-GAD-023-0199	Gracias a Dios	Puerto Lempira	Suhi	Resting collection	Culicidae	*Anopheles*	*Anopheles*	*crucians*	NEG
143	HND-2021-GAD-023-0200	Gracias a Dios	Puerto Lempira	Suhi	Resting collection	Culicidae	*Anopheles*	*Anopheles*	*vestitipennis*	NEG
144	HND-2021-GAD-023-0201	Gracias a Dios	Puerto Lempira	Suhi	Resting collection	Culicidae	*Anopheles*	*Anopheles*	*crucians*	NEG
145	HND-2021-GAD-023-0202	Gracias a Dios	Puerto Lempira	Suhi	Resting collection	Culicidae	*Anopheles*	*Anopheles*	*neomaculipalpus*	NEG
146	HND-2021-GAD-023-0203	Gracias a Dios	Puerto Lempira	Suhi	Resting collection	Culicidae	*Anopheles*	*Nyssorhyncus*	*albimanus*	NEG
147	HND-2021-GAD-023-0204	Gracias a Dios	Puerto Lempira	Suhi	Resting collection	Culicidae	*Anopheles*	*Nyssorhyncus*	*albimanus*	Not processed
148	HND-2021-GAD-023-0205	Gracias a Dios	Puerto Lempira	Suhi	Resting collection	Culicidae	*Anopheles*	*Nyssorhyncus*	*albimanus*	Not processed
149	HND-2021-GAD-023-0206	Gracias a Dios	Puerto Lempira	Suhi	Resting collection	Culicidae	*Anopheles*	*Anopheles*	*neomaculipalpus*	NEG
150	HND-2021-GAD-023-0207	Gracias a Dios	Puerto Lempira	Suhi	Resting collection	Culicidae	*Anopheles*	*Nyssorhyncus*	*argyritarsis*	NEG
151	HND-2021-GAD-023-0208	Gracias a Dios	Puerto Lempira	Suhi	Resting collection	Culicidae	*Anopheles*	*Nyssorhyncus*	*albimanus*	Not processed
152	HND-2021-GAD-023-0209	Gracias a Dios	Puerto Lempira	Suhi	Resting collection	Culicidae	*Anopheles*	*Nyssorhyncus*	*albimanus*	Not processed
153	HND-2021-GAD-023-0210	Gracias a Dios	Puerto Lempira	Suhi	Resting collection	Culicidae	*Anopheles*	*Nyssorhyncus*	*albimanus*	Not processed
154	HND-2021-GAD-023-0211	Gracias a Dios	Puerto Lempira	Suhi	Resting collection	Culicidae	*Anopheles*	*Nyssorhyncus*	*albimanus*	Not processed
155	HND-2021-GAD-023-0212	Gracias a Dios	Puerto Lempira	Suhi	Resting collection	Culicidae	*Anopheles*	*Nyssorhyncus*	*albimanus*	Not processed
156	HND-2021-GAD-023-0213	Gracias a Dios	Puerto Lempira	Suhi	Resting collection	Culicidae	*Anopheles*	*Nyssorhyncus*	*albimanus*	NEG
157	HND-2021-GAD-023-0214	Gracias a Dios	Puerto Lempira	Suhi	Resting collection	Culicidae	*Anopheles*	*Nyssorhyncus*	*albimanus*	NEG
158	HND-2021-GAD-023-0215	Gracias a Dios	Puerto Lempira	Suhi	Resting collection	Culicidae	*Anopheles*	*Nyssorhyncus*	*albimanus*	NEG

159	HND-2021-GAD-023-0216	Gracias a Dios	Puerto Lempira	Suhi	Resting collection	Culicidae	*Anopheles*	*Nyssorhyncus*	*albimanus*	NEG
160	HND-2021-GAD-023-0217	Gracias a Dios	Puerto Lempira	Suhi	Resting collection	Culicidae	*Anopheles*	*Nyssorhyncus*	*argyritarsis*	NEG
161	HND-2021-GAD-023-0218	Gracias a Dios	Puerto Lempira	Suhi	Resting collection	Culicidae	*Anopheles*	*Nyssorhyncus*	*albimanus*	NEG
162	HND-2021-GAD-023-0219	Gracias a Dios	Puerto Lempira	Suhi	Resting collection	Culicidae	*Anopheles*	*Nyssorhyncus*	*albimanus*	NEG
163	HND-2021-GAD-023-0220	Gracias a Dios	Puerto Lempira	Suhi	Resting collection	Culicidae	*Anopheles*	*Nyssorhyncus*	*albimanus*	NEG
164	HND-2021-GAD-023-0221	Gracias a Dios	Puerto Lempira	Suhi	Resting collection	Culicidae	*Anopheles*	*Nyssorhyncus*	*albimanus*	NEG
165	HND-2021-GAD-023-0222	Gracias a Dios	Puerto Lempira	Suhi	Resting collection	Culicidae	*Anopheles*	*Nyssorhyncus*	*albimanus*	NEG
166	HND-2021-GAD-023-0224	Gracias a Dios	Puerto Lempira	Suhi	Resting collection	Culicidae	*Anopheles*	*Nyssorhyncus*	*albimanus*	NEG
167	HND-2021-GAD-023-0225	Gracias a Dios	Puerto Lempira	Suhi	Resting collection	Culicidae	*Anopheles*	*Nyssorhyncus*	*albimanus*	NEG
168	HND-2021-GAD-023-0226	Gracias a Dios	Puerto Lempira	Suhi	Resting collection	Culicidae	*Anopheles*	*Nyssorhyncus*	*albimanus*	NEG
169	HND-2021-GAD-023-0227	Gracias a Dios	Puerto Lempira	Suhi	Resting collection	Culicidae	*Anopheles*	*Nyssorhyncus*	*albimanus*	NEG
170	HND-2021-GAD-023-0228	Gracias a Dios	Puerto Lempira	Suhi	Resting collection	Culicidae	*Anopheles*	*Nyssorhyncus*	*albimanus*	NEG
171	HND-2021-GAD-023-0229	Gracias a Dios	Puerto Lempira	Suhi	Resting collection	Culicidae	*Anopheles*	*Nyssorhyncus*	*albimanus*	NEG
172	HND-2021-GAD-023-0230	Gracias a Dios	Puerto Lempira	Suhi	Resting collection	Culicidae	*Anopheles*	*Nyssorhyncus*	*albimanus*	NEG
173	HND-2021-GAD-023-0231	Gracias a Dios	Puerto Lempira	Suhi	Resting collection	Culicidae	*Anopheles*	*Nyssorhyncus*	*albimanus*	NEG
174	HND-2021-GAD-023-0232	Gracias a Dios	Puerto Lempira	Suhi	Resting collection	Culicidae	*Anopheles*	*Nyssorhyncus*	*albimanus*	NEG
175	HND-2021-GAD-023-0233	Gracias a Dios	Puerto Lempira	Suhi	Resting collection	Culicidae	*Anopheles*	*Nyssorhyncus*	*albimanus*	NEG
176	HND-2021-GAD-023-0234	Gracias a Dios	Puerto Lempira	Suhi	Resting collection	Culicidae	*Anopheles*	*Nyssorhyncus*	*albimanus*	NEG
177	HND-2021-GAD-023-0235	Gracias a Dios	Puerto Lempira	Suhi	Resting collection	Culicidae	*Anopheles*	*Nyssorhyncus*	*albimanus*	NEG
178	HND-2021-GAD-023-0236	Gracias a Dios	Puerto Lempira	Suhi	Resting collection	Culicidae	*Anopheles*	*Nyssorhyncus*	*albimanus*	NEG
179	HND-2021-GAD-023-0237	Gracias a Dios	Puerto Lempira	Suhi	Resting collection	Culicidae	*Anopheles*	*Nyssorhyncus*	*albimanus*	NEG
180	HND-2021-GAD-023-0238	Gracias a Dios	Puerto Lempira	Suhi	Resting collection	Culicidae	*Anopheles*	*Nyssorhyncus*	*albimanus*	NEG
181	HND-2021-GAD-023-0239	Gracias a Dios	Puerto Lempira	Suhi	Resting collection	Culicidae	*Anopheles*	*Nyssorhyncus*	*albimanus*	NEG
182	HND-2021-GAD-023-0240	Gracias a Dios	Puerto Lempira	Suhi	Resting collection	Culicidae	*Anopheles*	*Nyssorhyncus*	*albimanus*	NEG
183	HND-2021-GAD-023-0241	Gracias a Dios	Puerto Lempira	Suhi	Resting collection	Culicidae	*Anopheles*	*Nyssorhyncus*	*albimanus*	NEG
184	HND-2021-GAD-023-0242	Gracias a Dios	Puerto Lempira	Suhi	Resting collection	Culicidae	*Anopheles*	*Anopheles*	*crucians*	NEG
185	HND-2021-GAD-023-0243	Gracias a Dios	Puerto Lempira	Suhi	Resting collection	Culicidae	*Anopheles*	*Anopheles*	*crucians*	NEG

186	HND-2021-GAD-023-0244	Gracias a Dios	Puerto Lempira	Suhi	Resting collection	Culicidae	*Anopheles*	*Anopheles*	*crucians*	NEG
187	HND-2021-GAD-023-0245	Gracias a Dios	Puerto Lempira	Suhi	Resting collection	Culicidae	*Anopheles*	*Nyssorhyncus*	*albimanus*	NEG
188	HND-2021-GAD-023-0254	Gracias a Dios	Puerto Lempira	Suhi	Resting collection	Culicidae	*Anopheles*	*Anopheles*	*neomaculipalpus*	NEG
189	HND-2021-GAD-023-0255	Gracias a Dios	Puerto Lempira	Suhi	Resting collection	Culicidae	*Anopheles*	*Anopheles*	*neomaculipalpus*	NEG
190	HND-2021-GAD-023-0256	Gracias a Dios	Puerto Lempira	Suhi	Resting collection	Culicidae	*Anopheles*	*Anopheles*	*punctimacula*	NEG
191	HND-2021-GAD-023-0257	Gracias a Dios	Puerto Lempira	Suhi	Resting collection	Culicidae	*Anopheles*	*Anopheles*	*neomaculipalpus*	NEG
192	HND-2021-GAD-023-0259	Gracias a Dios	Puerto Lempira	Suhi	Resting collection	Culicidae	*Anopheles*	*Anopheles*	*neomaculipalpus*	NEG
193	HND-2021-GAD-023-0260	Gracias a Dios	Puerto Lempira	Suhi	Resting collection	Culicidae	*Anopheles*	*Anopheles*	*neomaculipalpus*	NEG
194	HND-2021-GAD-023-0261	Gracias a Dios	Puerto Lempira	Suhi	Resting collection	Culicidae	*Anopheles*	*Nyssorhyncus*	*albimanus*	NEG
195	HND-2021-GAD-023-0262	Gracias a Dios	Puerto Lempira	Suhi	Resting collection	Culicidae	*Anopheles*	*Nyssorhyncus*	*albimanus*	Not processed
196	HND-2021-GAD-023-0263	Gracias a Dios	Puerto Lempira	Suhi	Resting collection	Culicidae	*Anopheles*	*Nyssorhyncus*	*albimanus*	NEG
197	HND-2021-GAD-023-0264	Gracias a Dios	Puerto Lempira	Suhi	Resting collection	Culicidae	*Anopheles*	*Nyssorhyncus*	*albimanus*	Not processed
198	HND-2021-GAD-023-0266	Gracias a Dios	Puerto Lempira	Suhi	Resting collection	Culicidae	*Anopheles*	*Nyssorhyncus*	*albimanus*	Not processed
199	HND-2021-GAD-023-0267	Gracias a Dios	Puerto Lempira	Suhi	Resting collection	Culicidae	*Anopheles*	*Nyssorhyncus*	*albimanus*	Not processed
200	HND-2021-GAD-023-0268	Gracias a Dios	Puerto Lempira	Suhi	Resting collection	Culicidae	*Anopheles*	*Nyssorhyncus*	*albimanus*	Not processed
201	HND-2021-GAD-023-0269	Gracias a Dios	Puerto Lempira	Suhi	Resting collection	Culicidae	*Anopheles*	*Nyssorhyncus*	*albimanus*	Not processed
202	HND-2021-GAD-023-0270	Gracias a Dios	Puerto Lempira	Suhi	Resting collection	Culicidae	*Anopheles*	*Nyssorhyncus*	*albimanus*	Not processed
203	HND-2021-GAD-023-0271	Gracias a Dios	Puerto Lempira	Suhi	Resting collection	Culicidae	*Anopheles*	*Nyssorhyncus*	*albimanus*	Not processed
204	HND-2021-GAD-023-0272	Gracias a Dios	Puerto Lempira	Suhi	Resting collection	Culicidae	*Anopheles*	*Nyssorhyncus*	*albimanus*	Not processed
205	HND-2021-GAD-023-0273	Gracias a Dios	Puerto Lempira	Suhi	Resting collection	Culicidae	*Anopheles*	*Nyssorhyncus*	*albimanus*	Not processed
206	HND-2021-GAD-023-0276	Gracias a Dios	Puerto Lempira	Suhi	Resting collection	Culicidae	*Anopheles*	*Anopheles*	*crucians*	NEG
207	HND-2021-GAD-023-0277	Gracias a Dios	Puerto Lempira	Suhi	Resting collection	Culicidae	*Anopheles*	*Nyssorhyncus*	*albimanus*	Not processed
208	HND-2021-GAD-023-0278	Gracias a Dios	Puerto Lempira	Suhi	Resting collection	Culicidae	*Anopheles*	*Nyssorhyncus*	*albimanus*	Not processed
209	HND-2021-GAD-023-0279	Gracias a Dios	Puerto Lempira	Suhi	Resting collection	Culicidae	*Anopheles*	*Nyssorhyncus*	*albimanus*	Not processed
210	HND-2021-GAD-023-0280	Gracias a Dios	Puerto Lempira	Suhi	Resting collection	Culicidae	*Anopheles*	*Nyssorhyncus*	*albimanus*	Not processed
211	HND-2021-GAD-023-0281	Gracias a Dios	Puerto Lempira	Suhi	Resting collection	Culicidae	*Anopheles*	*Nyssorhyncus*	*argyritarsis*	NEG
212	HND-2018-COM-024-0282	Comayagua	Comayagua		Shannon trap	Culicidae	*Anopheles*	*Nyssorhyncus*	*albimanus*	NEG

213	HND-2018-COM-024-0283	Comayagua	Comayagua		CDC Light Trap	Culicidae	*Anopheles*	*Nyssorhyncus*	*albimanus*	NEG
214	HND-2018-COM-024-0284	Comayagua	Comayagua		CDC Light Trap	Culicidae	*Anopheles*	*Nyssorhyncus*	*albimanus*	NEG
215	HND-2018-COM-024-0285	Comayagua	Comayagua		CDC Light Trap	Culicidae	*Anopheles*	*Nyssorhyncus*	*albimanus*	NEG
216	HND-2018-COM-024-0286	Comayagua	La Libertad	Gracias a Dios	Resting collection	Culicidae	*Anopheles*	*Anopheles*	*pseudopunctipennis*	NEG
217	HND-2018-COM-024-0287	Comayagua	La Libertad	Gracias a Dios	Resting collection	Culicidae	*Anopheles*	*Anopheles*	*pseudopunctipennis*	NEG
218	HND-2019-COM-024-0288	Comayagua	Comayagua	Aldea Fátima, San Jeronimo	Resting collection	Culicidae	*Anopheles*	*Anopheles*	*pseudopunctipennis*	NEG
219	HND-2019-COM-024-0289	Comayagua	Comayagua	Aldea Fátima, San Jeronimo	Resting collection	Culicidae	*Anopheles*	*Anopheles*	*pseudopunctipennis*	NEG
220	HND-2019-COM-024-0290	Comayagua	Comayagua	Aldea Fátima, San Jeronimo	Resting collection	Culicidae	*Anopheles*	*Anopheles*	*pseudopunctipennis*	NEG
221	HND-2019-COM-024-0291	Comayagua	Comayagua	Aldea Fátima, San Jeronimo	Resting collection	Culicidae	*Anopheles*	*Anopheles*	*pseudopunctipennis*	NEG
222	HND-2019-COM-024-0292	Comayagua	Comayagua	Aldea Fátima, San Jeronimo	Resting collection	Culicidae	*Anopheles*	*Anopheles*	*pseudopunctipennis*	NEG
223	HND-2019-COM-024-0293	Comayagua	Comayagua	Aldea Fátima, San Jeronimo	Resting collection	Culicidae	*Anopheles*	*Anopheles*	*pseudopunctipennis*	NEG
224	HND-2019-COM-024-0294	El Paraíso	Morocelí	Barrio El Jicaro, Aldea Guadalajara	CDC Light Trap	Culicidae	*Anopheles*	*Nyssorhyncus*	*albimanus*	NEG
225	HND-2019-COM-024-0295	El Paraíso	Morocelí	Barrio El Jicaro, Aldea Guadalajara	Resting collection	Culicidae	*Anopheles*	*Nyssorhyncus*	*albimanus*	NEG
226	HND-2019-COM-024-0296	El Paraíso	Morocelí	Barrio El Jicaro, Aldea Guadalajara	Resting collection	Culicidae	*Anopheles*	*Nyssorhyncus*	*albimanus*	NEG
227	HND-2019-COM-024-0297	El Paraíso	Morocelí	Barrio El Jicaro, Aldea Guadalajara	Resting collection	Culicidae	*Anopheles*	*Nyssorhyncus*	*albimanus*	NEG
228	HND-2019-COM-024-0298	El Paraíso	Morocelí	Barrio El Jicaro, Aldea Guadalajara	Resting collection	Culicidae	*Anopheles*	*Nyssorhyncus*	*albimanus*	NEG
229	HND-2019-COM-024-0299	El Paraíso	Morocelí	Barrio El Jicaro, Aldea Guadalajara	Resting collection	Culicidae	*Anopheles*	*Nyssorhyncus*	*albimanus*	NEG
230	HND-2019-COM-024-0300	El Paraíso	Morocelí	Barrio El Jicaro, Aldea Guadalajara	Resting collection	Culicidae	*Anopheles*	*Nyssorhyncus*	*albimanus*	NEG
231	HND-2019-COM-024-0301	El Paraíso	Morocelí	Barrio El Jicaro, Aldea Guadalajara	Resting collection	Culicidae	*Anopheles*	*Nyssorhyncus*	*albimanus*	NEG
232	HND-2019-COM-024-0302	El Paraíso	Morocelí	Barrio El Jicaro, Aldea Guadalajara	Resting collection	Culicidae	*Anopheles*	*Nyssorhyncus*	*albimanus*	NEG
233	HND-2019-COM-024-0303	El Paraíso	Morocelí	Barrio El Jicaro, Aldea Guadalajara	Resting collection	Culicidae	*Anopheles*	*Nyssorhyncus*	*albimanus*	NEG
234	HND-2019-COM-024-0304	El Paraíso	Morocelí	Barrio El Jicaro, Aldea Guadalajara	Resting collection	Culicidae	*Anopheles*	*Nyssorhyncus*	*albimanus*	NEG
235	HND-2019-COM-024-0305	El Paraíso	Morocelí	Barrio El Jicaro, Aldea Guadalajara	Resting collection	Culicidae	*Anopheles*	*Nyssorhyncus*	*albimanus*	NEG

236	HND-2019-COM-024-0306	El Paraíso	Morocelí	Barrio El Jicaro, Aldea Guadalajara	Resting collection	Culicidae	*Anopheles*	*Nyssorhyncus*	*albimanus*	NEG
237	HND-2019-COM-024-0307	El Paraíso	Morocelí	Barrio El Jicaro, Aldea Guadalajara	Resting collection	Culicidae	*Anopheles*	*Nyssorhyncus*	*albimanus*	NEG
238	HND-2019-COM-024-0308	El Paraíso	Morocelí	Barrio El Jicaro, Aldea Guadalajara	Resting collection	Culicidae	*Anopheles*	*Nyssorhyncus*	*albimanus*	NEG
239	HND-2019-COM-024-0309	El Paraíso	Morocelí	Barrio El Jicaro, Aldea Guadalajara	Resting collection	Culicidae	*Anopheles*	*Nyssorhyncus*	*albimanus*	NEG
240	HND-2019-COM-024-0310	El Paraíso	Morocelí	Barrio El Jicaro, Aldea Guadalajara	Resting collection	Culicidae	*Anopheles*	*Nyssorhyncus*	*albimanus*	NEG
241	HND-2019-COM-024-0311	El Paraíso	Morocelí	Barrio El Jicaro, Aldea Guadalajara	Resting collection	Culicidae	*Anopheles*	*Nyssorhyncus*	*albimanus*	NEG
242	HND-2019-COM-024-0312	El Paraíso	Morocelí	Barrio El Jicaro, Aldea Guadalajara	Resting collection	Culicidae	*Anopheles*	*Nyssorhyncus*	*albimanus*	NEG
243	HND-2019-COM-024-0316	Comayagua	Comayagua		CDC Light Trap	Culicidae	*Anopheles*	*Nyssorhyncus*	*albimanus*	NEG
244	HND-2019-COM-024-0317	Comayagua	Comayagua		BG Sentinel	Culicidae	*Anopheles*	*Nyssorhyncus*	*albimanus*	NEG
245	HND-2020-COM-024-0318	Comayagua	Comayagua		CDC Light Trap	Culicidae	*Anopheles*	*Nyssorhyncus*	*albimanus*	NEG
246	HND-2020-COM-024-0321	Comayagua	Comayagua		CDC Light Trap	Culicidae	*Anopheles*	*Anopheles*	*pseudopunctipennis*	NEG
247	HND-2021-COM-024-0322	Cortés	Santa Cruz de Yojoa	San Antonio de Yure	CDC Light Trap	Culicidae	*Anopheles*	*Nyssorhyncus*	*albimanus*	NEG
248	HND-2021-COM-024-0323	Comayagua	San Jose de C.	Barrio Montefresco	CDC Light Trap	Culicidae	*Anopheles*	*Nyssorhyncus*	*argyritarsis*	NEG
249	HND-2021-COM-024-0324	Comayagua	San Jose de C.	Barrio Montefresco	CDC Light Trap	Culicidae	*Anopheles*	*Nyssorhyncus*	*argyritarsis*	NEG
250	HND-2021-COM-024-0325	Comayagua	San Jose de C.	Barrio Montefresco	CDC Light Trap	Culicidae	*Anopheles*	*Nyssorhyncus*	*argyritarsis*	NEG
251	HND-2021-COM-024-0326	Comayagua	San Jose de C.	Barrio Montefresco	CDC Light Trap	Culicidae	*Anopheles*	*Nyssorhyncus*	*argyritarsis*	NEG
252	HND-2021-COM-024-0327	Comayagua	San Jose de C.	Barrio Montefresco	CDC Light Trap	Culicidae	*Anopheles*	*Nyssorhyncus*	*argyritarsis*	NEG
253	HND-2021-COM-024-0328	Comayagua	San Jose de C.	Barrio Montefresco	CDC Light Trap	Culicidae	*Anopheles*	*Nyssorhyncus*	*argyritarsis*	NEG
254	HND-2021-COM-024-0329	Comayagua	San Jose de C.	Barrio Montefresco	CDC Light Trap	Culicidae	*Anopheles*	*Nyssorhyncus*	*argyritarsis*	NEG
255	HND-2021-COM-024-0330	Cortés	Pimienta	Colonia Barahona	CDC Light Trap	Culicidae	*Anopheles*	*Nyssorhyncus*	*albimanus*	NEG
256	HND-2021-COM-024-0331	Cortés	Pimienta	Colonia Barahona	CDC Light Trap	Culicidae	*Anopheles*	*Nyssorhyncus*	*albimanus*	NEG
257	HND-2021-IB-025-0332	Islas de la Bahía	Roatan	Sandy Bay	Resting collection	Culicidae	*Anopheles*	*Nyssorhyncus*	*albimanus*	NEG
258	HND-2021-IB-025-0333	Islas de la Bahía	Roatan	Sandy Bay	Resting collection	Culicidae	*Anopheles*	*Nyssorhyncus*	*albimanus*	NEG
259	HND-2021-IB-025-0334	Islas de la Bahía	Roatan	Sandy Bay	Resting collection	Culicidae	*Anopheles*	*Nyssorhyncus*	*albimanus*	NEG
260	HND-2021-IB-025-0335	Islas de la Bahía	Roatan	Sandy Bay	Resting collection	Culicidae	*Anopheles*	*Nyssorhyncus*	*albimanus*	NEG

261	HND-2021-IB-025-0336	Islas de la Bahía	Roatan	Sandy Bay	Resting collection	Culicidae	*Anopheles*	*Nyssorhyncus*	*albimanus*	NEG
262	HND-2021-IB-025-0337	Islas de la Bahía	Roatan	Sandy Bay	Resting collection	Culicidae	*Anopheles*	*Nyssorhyncus*	*albimanus*	NEG
263	HND-2021-IB-025-0338	Islas de la Bahía	Roatan	Sandy Bay	Resting collection	Culicidae	*Anopheles*	*Nyssorhyncus*	*albimanus*	NEG
264	HND-2021-IB-025-0339	Islas de la Bahía	Roatan	Sandy Bay	Resting collection	Culicidae	*Anopheles*	*Nyssorhyncus*	*albimanus*	NEG
265	HND-2021-IB-025-0340	Islas de la Bahía	Roatan	Sandy Bay	Resting collection	Culicidae	*Anopheles*	*Nyssorhyncus*	*albimanus*	NEG
266	HND-2021-IB-025-0341	Islas de la Bahía	Roatan	Sandy Bay	Resting collection	Culicidae	*Anopheles*	*Nyssorhyncus*	*albimanus*	NEG
267	HND-2021-IB-025-0342	Islas de la Bahía	Roatan	Sandy Bay	Resting collection	Culicidae	*Anopheles*	*Nyssorhyncus*	*albimanus*	NEG
268	HND-2021-IB-025-0343	Islas de la Bahía	Roatan	Sandy Bay	Resting collection	Culicidae	*Anopheles*	*Nyssorhyncus*	*albimanus*	NEG
269	HND-2021-IB-025-0344	Islas de la Bahía	Roatan	Sandy Bay	Resting collection	Culicidae	*Anopheles*	*Nyssorhyncus*	*albimanus*	NEG
270	HND-2021-IB-025-0345	Islas de la Bahía	Roatan	Sandy Bay	Resting collection	Culicidae	*Anopheles*	*Nyssorhyncus*	*albimanus*	NEG
271	HND-2021-IB-025-0346	Islas de la Bahía	Roatan	Sandy Bay	Resting collection	Culicidae	*Anopheles*	*Nyssorhyncus*	*albimanus*	NEG
272	HND-2021-IB-025-0347	Islas de la Bahía	Roatan	Sandy Bay	Resting collection	Culicidae	*Anopheles*	*Nyssorhyncus*	*albimanus*	NEG
273	HND-2021-IB-025-0348	Islas de la Bahía	Roatan	Sandy Bay	Resting collection	Culicidae	*Anopheles*	*Nyssorhyncus*	*albimanus*	NEG
274	HND-2021-IB-025-0349	Islas de la Bahía	Roatan	Sandy Bay	Resting collection	Culicidae	*Anopheles*	*Nyssorhyncus*	*albimanus*	NEG
275	HND-2021-IB-025-0350	Islas de la Bahía	Roatan	Sandy Bay	Resting collection	Culicidae	*Anopheles*	*Nyssorhyncus*	*albimanus*	Not processed
276	HND-2021-IB-025-0351	Islas de la Bahía	Roatan	Sandy Bay	Resting collection	Culicidae	*Anopheles*	*Nyssorhyncus*	*albimanus*	Not processed
277	HND-2021-IB-025-0353	Islas de la Bahía	Roatan	Sandy Bay	Resting collection	Culicidae	*Anopheles*	*Nyssorhyncus*	*albimanus*	Not processed
278	HND-2021-IB-025-0354	Islas de la Bahía	Roatan	Sandy Bay	Resting collection	Culicidae	*Anopheles*	*Nyssorhyncus*	*albimanus*	Not processed
279	HND-2021-IB-025-0355	Islas de la Bahía	Roatan	Sandy Bay	Resting collection	Culicidae	*Anopheles*	*Nyssorhyncus*	*albimanus*	NEG
280	HND-2021-IB-025-0356	Islas de la Bahía	Roatan	Sandy Bay	Resting collection	Culicidae	*Anopheles*	*Nyssorhyncus*	*albimanus*	NEG
281	HND-2021-IB-025-0357	Islas de la Bahía	Roatan	Sandy Bay	Resting collection	Culicidae	*Anopheles*	*Nyssorhyncus*	*albimanus*	NEG
282	HND-2021-IB-025-0358	Islas de la Bahía	Roatan	Sandy Bay	Resting collection	Culicidae	*Anopheles*	*Nyssorhyncus*	*albimanus*	NEG
283	HND-2021-IB-025-0359	Islas de la Bahía	Roatan	Sandy Bay	Resting collection	Culicidae	*Anopheles*	*Nyssorhyncus*	*albimanus*	NEG
284	HND-2021-IB-025-0360	Islas de la Bahía	Roatan	Sandy Bay	Resting collection	Culicidae	*Anopheles*	*Nyssorhyncus*	*albimanus*	NEG
285	HND-2021-IB-025-0361	Islas de la Bahía	Roatan	Sandy Bay	Resting collection	Culicidae	*Anopheles*	*Nyssorhyncus*	*albimanus*	NEG
286	HND-2021-IB-025-0362	Islas de la Bahía	Roatan	Sandy Bay	Resting collection	Culicidae	*Anopheles*	*Nyssorhyncus*	*albimanus*	NEG
287	HND-2021-IB-025-0363	Islas de la Bahía	Roatan	Sandy Bay	Resting collection	Culicidae	*Anopheles*	*Nyssorhyncus*	*albimanus*	NEG

288	HND-2021-IB-025-0364	Islas de la Bahía	Roatan	Sandy Bay	Resting collection	Culicidae	*Anopheles*	*Nyssorhyncus*	*albimanus*	NEG
289	HND-2019-COM-024-0289	Comayagua	Comayagua	Aldea Fátima, San Jeronimo	Resting collection	Culicidae	*Anopheles*	*Anopheles*	*pseudopunctipennis*	NEG
290	HND-2019-COM-024-0290	Comayagua	Comayagua	Aldea Fátima, San Jeronimo	Resting collection	Culicidae	*Anopheles*	*Anopheles*	*pseudopunctipennis*	NEG
291	HND-2019-COM-024-0291	Comayagua	Comayagua	Aldea Fátima, San Jeronimo	Resting collection	Culicidae	*Anopheles*	*Anopheles*	*pseudopunctipennis*	NEG
292	HND-2019-COM-024-0292	Comayagua	Comayagua	Aldea Fátima, San Jeronimo	Resting collection	Culicidae	*Anopheles*	*Anopheles*	*pseudopunctipennis*	NEG
293	HND-2019-COM-024-0293	Comayagua	Comayagua	Aldea Fátima, San Jeronimo	Resting collection	Culicidae	*Anopheles*	*Anopheles*	*pseudopunctipennis*	NEG
294	HND-2019-COM-024-0294	El Paraíso	Morocelí	Barrio El Jicaro, Aldea Guadalajara	CDC Light Trap	Culicidae	*Anopheles*	*Nyssorhyncus*	*albimanus*	NEG
295	HND-2019-COM-024-0295	El Paraíso	Morocelí	Barrio El Jicaro, Aldea Guadalajara	Resting collection	Culicidae	*Anopheles*	*Nyssorhyncus*	*albimanus*	NEG
296	HND-2019-COM-024-0296	El Paraíso	Morocelí	Barrio El Jicaro, Aldea Guadalajara	Resting collection	Culicidae	*Anopheles*	*Nyssorhyncus*	*albimanus*	NEG
297	HND-2019-COM-024-0297	El Paraíso	Morocelí	Barrio El Jicaro, Aldea Guadalajara	Resting collection	Culicidae	*Anopheles*	*Nyssorhyncus*	*albimanus*	NEG
298	HND-2019-COM-024-0298	El Paraíso	Morocelí	Barrio El Jicaro, Aldea Guadalajara	Resting collection	Culicidae	*Anopheles*	*Nyssorhyncus*	*albimanus*	NEG
299	HND-2019-COM-024-0299	El Paraíso	Morocelí	Barrio El Jicaro, Aldea Guadalajara	Resting collection	Culicidae	*Anopheles*	*Nyssorhyncus*	*albimanus*	NEG
300	HND-2019-COM-024-0300	El Paraíso	Morocelí	Barrio El Jicaro, Aldea Guadalajara	Resting collection	Culicidae	*Anopheles*	*Nyssorhyncus*	*albimanus*	NEG
301	HND-2019-COM-024-0301	El Paraíso	Morocelí	Barrio El Jicaro, Aldea Guadalajara	Resting collection	Culicidae	*Anopheles*	*Nyssorhyncus*	*albimanus*	NEG
302	HND-2019-COM-024-0302	El Paraíso	Morocelí	Barrio El Jicaro, Aldea Guadalajara	Resting collection	Culicidae	*Anopheles*	*Nyssorhyncus*	*albimanus*	NEG
319	HND-2020-COM-024-0319	Comayagua	Comayagua		BG Sentinel	Culicidae	*Anopheles*	*Nyssorhyncus*	*albimanus*	NEG
320	HND-2020-COM-024-0320	Comayagua	Comayagua		BG Sentinel	Culicidae	*Anopheles*	*Nyssorhyncus*	*albimanus*	NEG
321	HND-2020-COM-024-0321	Comayagua	Comayagua		CDC Light Trap	Culicidae	*Anopheles*	*Anopheles*	*pseudopunctipennis*	NEG
322	HND-2021-COM-024-0322	Cortés	Santa Cruz de Yojoa	San Antonio de Yure	CDC Light Trap	Culicidae	*Anopheles*	*Nyssorhyncus*	*albimanus*	NEG
323	HND-2021-COM-024-0323	Comayagua	San Jose de C.	Barrio Montefresco	CDC Light Trap	Culicidae	*Anopheles*	*Nyssorhyncus*	*argyritarsis*	NEG
324	HND-2021-COM-024-0324	Comayagua	San Jose de C.	Barrio Montefresco	CDC Light Trap	Culicidae	*Anopheles*	*Nyssorhyncus*	*argyritarsis*	NEG
325	HND-2021-COM-024-0325	Comayagua	San Jose de C.	Barrio Montefresco	CDC Light Trap	Culicidae	*Anopheles*	*Nyssorhyncus*	*argyritarsis*	NEG
326	HND-2021-COM-024-0326	Comayagua	San Jose de C.	Barrio Montefresco	CDC Light Trap	Culicidae	*Anopheles*	*Nyssorhyncus*	*argyritarsis*	NEG
327	HND-2021-COM-024-0327	Comayagua	San Jose de C.	Barrio Montefresco	CDC Light Trap	Culicidae	*Anopheles*	*Nyssorhyncus*	*argyritarsis*	NEG

328	HND-2021-COM-024-0328	Comayagua	San Jose de C.	Barrio Montefresco	CDC Light Trap	Culicidae	*Anopheles*	*Nyssorhyncus*	*argyritarsis*	NEG
329	HND-2021-COM-024-0329	Comayagua	San Jose de C.	Barrio Montefresco	CDC Light Trap	Culicidae	*Anopheles*	*Nyssorhyncus*	*argyritarsis*	NEG
330	HND-2021-COM-024-0330	Cortés	Pimienta	Colonia Barahona	CDC Light Trap	Culicidae	*Anopheles*	*Nyssorhyncus*	*albimanus*	NEG
331	HND-2021-COM-024-0331	Cortés	Pimienta	Colonia Barahona	CDC Light Trap	Culicidae	*Anopheles*	*Nyssorhyncus*	*albimanus*	NEG
